# Rubbing salt in the wound? A large-scale investigation into the effects of refactoring on security

**DOI:** 10.1007/s10664-023-10287-x

**Published:** 2023-05-24

**Authors:** Emanuele Iannone, Zadia Codabux, Valentina Lenarduzzi, Andrea De Lucia, Fabio Palomba

**Affiliations:** 1 SeSa Lab, University of Salerno, Via Giovanni Paolo II, 132, 84084 Fisciano, SA Italy; 2 University of Saskatchewan, Saskatoon, SK Canada; 3 University of Oulu, Pentti Kaiteran katu 1, 90570 Oulu, Finland

**Keywords:** Refactoring, Software security, Empirical SE

## Abstract

Software refactoring is a behavior-preserving activity to improve the source code quality without changing its external behavior. Unfortunately, it is often a manual and error-prone task that may induce regressions in the source code. Researchers have provided initial compelling evidence of the relation between refactoring and defects, yet little is known about how much it may impact software security. This paper bridges this knowledge gap by presenting a large-scale empirical investigation into the effects of refactoring on the security profile of applications. We conduct a three-level mining software repository study to establish the impact of 14 refactoring types on (i) security-related metrics, (ii) security technical debt, and (iii) the introduction of known vulnerabilities. The study covers 39 projects and a total amount of 7,708 refactoring commits. The key results show that refactoring has a limited connection to security. However, *Inline Method* and *Extract Interface* statistically contribute to improving some security aspects connected to encapsulating security-critical code components. *Extract Superclass* and *Pull Up Attribute* refactoring are commonly found in commits violating specific security best practices for writing secure code. Finally, *Extract Superclass* and *Extract & Move Method* refactoring tend to occur more often in commits contributing to the introduction of vulnerabilities. We conclude by distilling lessons learned and recommendations for researchers and practitioners.

## Introduction

In 1999, Fowler defined the term *“software refactoring”* to indicate the activities developers perform to improve the internal structure of source code without changing its external behavior (Martin and Kent 1999). Since then, the research community has investigated refactoring from multiple perspectives (Al Dallal and Abdin 2017; Azeem et al. 2019; de Paulo Sobrinho et al. 2018; Mens and Tourwé 2004), proposed novel recommendation systems to help developers refactor source code (Bavota et al. 2014; Terra et al. 2018; Tsantalis and Chatzigeorgiou 2009), empirically investigated why developers refactor source code (Bavota et al. 2015; Silva et al. 2016), studied the current barriers preventing refactoring in practice (Murphy-Hill and Black 2008; Kim et al. 2014; Sharma et al. 2015; Vassallo et al. 2018), and explored the effects of refactoring on source code dependability (Abid et al. 2020; Di Penta et al. 2020; Palomba et al. 2017; Stroggylos and Spinellis 2007).

One of the most worrisome results of these empirical analyses is that refactoring might induce defects. Bavota et al. (2012) and, more recently, Di Penta et al. (2020) have indeed shown that refactoring operations can induce faults in a non-negligible number of cases—this is likely due to refactoring operations done manually rather than supported by semi-automated tools (Kim et al. 2014).

This study builds on this line of research and investigates the relationship between refactoring and software security, defined as the property that allows the software to continue working correctly under potential risks due to external malicious attacks that may cause loss or harm (McGraw 2004). Our study is based on the assumption that refactoring operations performed by developers can lead to variations in the security level of an application. In the first place, this assumption is justified by early work studying the relation between refactoring and security measured in various ways. In particular, Abid et al. (2020) recently proposed a search-based security-aware refactoring recommender that suggests the refactoring operations to apply to obtain the best trade-off between code maintainability and security degradation. While the main focus of such work was the definition of a novel refactoring recommender, they also conducted a preliminary motivational analysis to correlate (i) the presence of 14 automatically-detectable refactoring types (Alizadeh et al. 2018) and (ii) the QMOOD metrics (Goyal and Joshi 2014) with eight data-access security indicators proposed in literature (Alshammari et al. 2009). The analysis considered a single snapshot of 30 open-source software systems and revealed that some refactoring types are negatively correlated to security—i.e., their application caused the worsening of certain security characteristics. Among their findings, they observed a negative correlation between the application of *Extract Superclass* refactoring operation (Martin and Kent 1999) and data-access security indicators—which is something we also observed in the context of our research. For instance, let us consider the case of project Conversations^1^ at the revision 2067b9bd, where the application of an *Extract Superclass* has led to the extraction of class XmppUri from class Invite. This refactoring caused the introduction of new security-sensitive attributes. Thus, it seems reasonable to believe that refactoring might impact an application’s security profile. Indeed, *Extract Superclass* revolves around modifying hierarchies to create a common superclass for a set of classes. By design, a superclass is more accessible than subclasses, which might expose previously hidden sensitive parts of the program, increasing the chances of introducing vulnerabilities. In a similar manner, Abid et al. (2020) found correlations between other refactoring operations (e.g., *Move Method* Martin and Kent 1999) and other security-related aspects.

Our research identifies a set of refactoring types whose application might actually lead to variations of the security level of the code being refactored—as detailed later in Section 2.3.1. In this sense, we build our empirical analysis upon logical reasoning, through which we hypothesized and verified the extent of the identified relations. For example, we hypothesize that the *Pull Up Attribute* refactoring (Martin and Kent 1999)—i.e., moving an attribute from a subclass to a superclass, changing its visibility and external accessibility—potentially leads to security drifts due to the wider exposition of the attribute. As an additional example, let us consider a case observed in the context of our research. This pertains to project batik^2^ at the revision 8309088a. The commit applied a great restructuring of the classes, as also pointed out by the commit message reported in the following:

“The deepest architectural change is a strong move towards tiling everything [...]”.

**Fig. 1:**
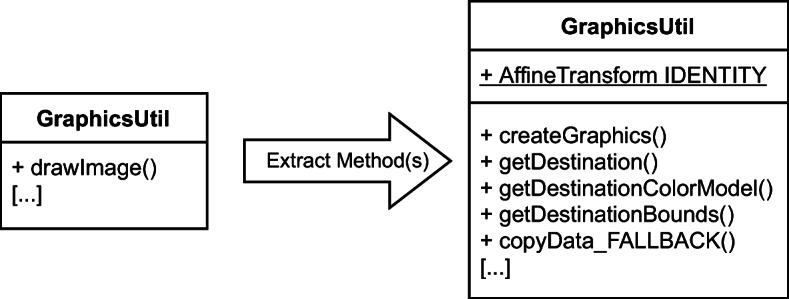
Simplified graphical representation of the changes applied to GraphicsUtil class at the revision 8309088a in project batik

Elaborating on the modifications performed in this commit, the GraphicsUtils class was affected by the various changes, being subject to several *Extract Method* refactoring operations. Specifically, the main restructuring involved the long method drawImage(), whose logic was decomposed into several smaller and more cohesive methods. In the version before the change, the drawImage() method allocated a new instance of the AffineTransform class each time it was called. Likely, this was judged as invalid, so making the commit’s author introduce a new class variable (i.e., a static class field) pointing to an instance of AffineTransform class named IDENTITY having public visibility. However, they likely ended up leaving it not-final, making it modifiable from any other class that has access to GraphicsUtils—i.e., potentially any project that includes batik library in their classpath. This scenario represents a pointless exposure of the class variable to any change, likely introducing bugs or even leaking information that should not be disclosed to clients. Figure 1 shows the GraphicsUtils class before and after the application of the multiple *Extract Method* refactoring operations.

Based on these observations and recognizing the significant research advances done by Abid et al. (2020), we aim to substantially enlarge the knowledge of the relation between refactoring and security, using different statistical methods and looking at different aspects characterizing software security. More specifically, we aimed at defining a *theory* that could provide quantitative indications of how different refactoring operations may impact security under different perspectives. Hence, we consider the change history information of 39 software projects and conduct a three-level quantitative analysis. We first assess the extent to which 14 refactoring types extracted by RefactoringMiner (Tsantalis et al. 2018) may affect the source code from a security perspective. As such, we measure the effects of refactoring on (i) a set of security metrics available in literature (Alshammari et al. 2009) and computed with a homemade tool, coined Surface (**S** ec**UR** ity **F** l**A** ws metri**C** s **E** xtractor), that we publicly release to the research community, and (ii) security-related technical debt, as computed by SonarQube.^3^ In doing so, we use similar statistical instruments as in previous studies investigating the relation between refactoring and source code quality (Bavota et al. 2015), program comprehension (Sellitto et al. 2022), and defects (Di Penta et al. 2020). In particular, statistical models specify mathematical relationships between one or more independent variables (in our case, the refactoring operations and a set of confounding variables) and dependent variables (in our case, the source code security level computed using security metrics and technical debt). As such, statistical modeling allows us to formally represent our theory (Adèr 2008), perfectly fitting the goals of our study. As the last part of the study, we verified how refactoring could lead to the introduction of known vulnerabilities mined from the National Vulnerability Database (NVD) (2023b). In this case, we first measured the number of times refactoring operations are performed in commits where known vulnerabilities are introduced; then, we conducted a finer-grained manual investigation to understand the extent to which refactoring operations are actually contributing to the introduction of vulnerabilities.

The key results of our investigation show a limited connection between refactoring and security. Indeed, we discover that most of the refactoring operations do not have a significant impact on any of the security perspectives considered. At the same time, we highlight some noticeable exceptions: *Inline Method* and *Extract Interface* are the refactoring operations that appear to be statistically significant when it turns to the improvement of some security aspects connected to encapsulation, while *Extract Superclass*, and *Pull Up Attribute* are linked to an increase of violations to certain security practices to write secure code. Furthermore, the *Extract Superclass* and *Extract & Move Method* refactoring types tend to occur more often in commits contributing to the introduction of real vulnerabilities. Based on our findings, we identify and distill a set of concrete issues and challenges that the refactoring community should face to better support developers. To sum up, this study provides the following contributions:

1. An evidence-based investigation into the relation between refactoring and security that targets the problem under three different perspectives, such as security metrics, security-related technical debt, and known vulnerabilities;
2. A research roadmap that researchers in the field can exploit to understand further the circumstances that lead refactoring to negatively affect security and provide automated support for practitioners;
3. An online appendix (Iannone et al. 2022) reporting all the data and scripts used in the study to allow researchers to replicate and conduct additional investigations.

### Structure of the Paper

Section 2 reports the methodology employed in the study, while Section 3 discusses the results achieved. In Section 4, we provide an overview of the main discussion points and implications of the results for the research community and practitioners. Section 5 reports on the threats that may have biased our findings. Section 6 discusses the related literature. Finally, Section 7 concludes the paper.

## Research Methodology

The *goal* of this study is to assess the relation between refactoring and security, with the *purpose* of understanding how refactoring operations applied by developers introduce security threats. The *perspective* is of both researchers and practitioners: the former are interested in understanding which additional support developers would require when performing refactoring; the latter are interested in evaluating the potential consequences of refactoring on source code dependability. Our study was designed based on the guidelines proposed by Runeson and Host (Per and Martin 2009) and follows the ACM/SIGSOFT Empirical Standards recently introduced and discussed by Ralph et al. (2021).^4^

### Research Questions and Methodological Overview

The empirical study is based on three levels of analysis. Following the preliminary investigation by Abid et al. (2020)—who observed a correlation between the amount of refactoring operations applied and security metrics—we aimed at assessing the security implications of refactoring operations on security indicators in an effort to provide insights into the potential compromise a developer should pay attention to while improving source code quality.

We start facing this research objective using two analyses: the first focused on security-related metrics that indicate portions of source code whose characteristics may lead the code to be more exposed to security risks (Alshammari et al. 2009); the second targeting technical debt (Curtis et al. 2012) that highlights the design and implementation issues that might represent exploitable security flaws. These two analyses are by nature complementary: security-related metrics focus on weak constructs implemented in the source code, while security technical debt measures on higher-level poor design or implementation solutions that might possibly impact the security profile of an application. As further explained in Section 2.3, we conducted these analyses by measuring developers’ activities, and their implications for source code security by running tools and analyses on commits where refactoring has been applied. These goals led to the formulation of the following two research questions: 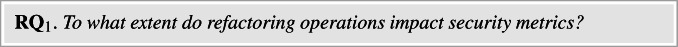

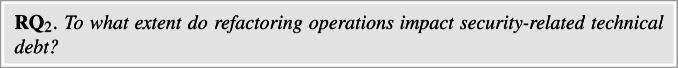


**Fig. 2:**
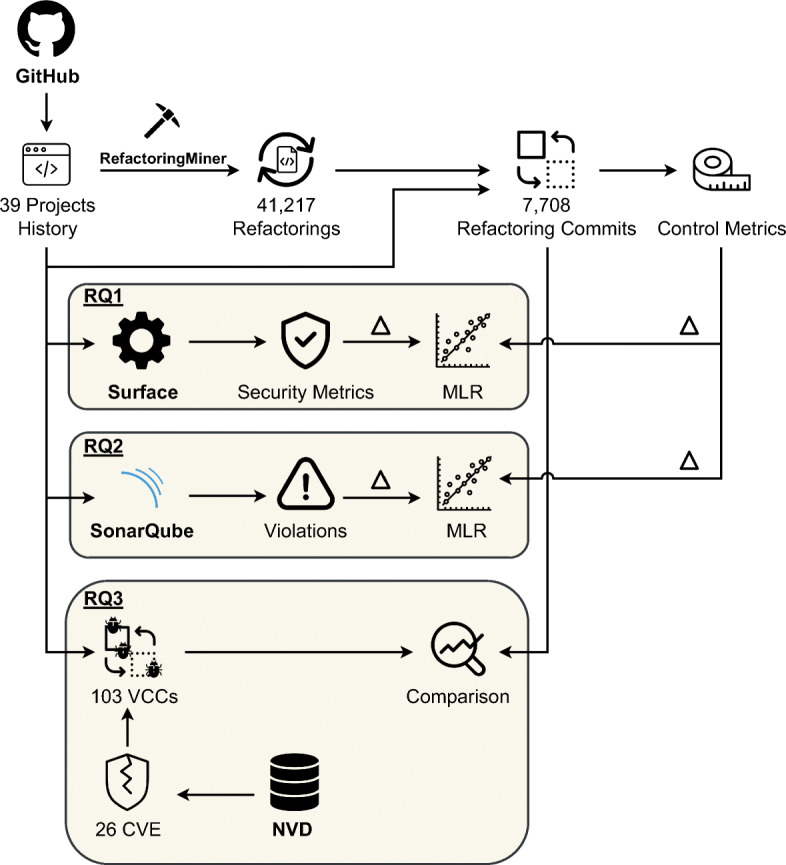
Methodological steps employed in our study

While the first two research questions allowed us to uncover possible negative effects given the application of refactoring operations on security, these were not sufficient nor comprehensive. Both security-related metrics and technical debt focus on *potential* risks for source code security; yet, this does not still clarify if and how refactoring has an impact on the introduction of *real* security threats. For this reason, we continued our empirical investigation by assessing how the application of refactoring operations over the change history of software projects leads to the introduction of known software vulnerabilities. This reasoning led to our last research question: 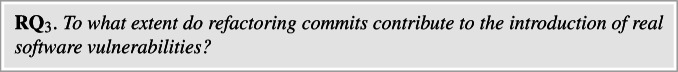


The study can be configured as a quantitative investigation (Sukamolson 2007) where we seek to find statistically significant findings from a large amount of data. While the next sections detail the data collection and analysis procedures used to address our research questions, Fig. 2 overviews the methodology employed in our study. In short, when addressing **RQ**_1_ and **RQ**_2_ we first run three tools, namely an automated refactoring detector called RefactoringMiner (Tsantalis et al. 2018), a security metric tool named Surface, and a static code analyzer called SonarQube over all the commits of the considered projects. Afterward, we use the data collected to compute the difference in terms of security metrics and debt between the refactoring commits and their predecessors to indicate how the refactoring operations have changed these measures. Finally, the variation of security metrics and debt were used as dependent variables of Multinomial Log-Linear regression models (Theil 1969) that allowed us to identify which refactoring operations are statistically related to their increase or decrease while controlling for factors like complexity, lines of code, and code churn.

As for **RQ**_3_, we mined the vulnerability-fixing commits of known vulnerabilities affecting the software projects considered in our study and available on a public dataset of vulnerabilities, namely, the National Vulnerability Database (NVD) (2023b). Then, we employed an automated mechanism based on the SZZ algorithm (Sliwerski et al. 2005) to identify the commits responsible for the introduction of those known vulnerabilities and combined this information with the one from RefactoringMiner to obtain the number of times refactoring operations are likely to have contributed to a known vulnerability. A manual qualitative investigation later contextualized the statistical analyses to understand further and discuss the quantitative results. The detailed methodological steps adopted to collect the described data are reported in Section 2.3.

### Context of the Study

The *context* of the study was composed of open-source software projects and, in particular, their change history information. In this respect, we exploited the *Technical Debt Dataset* (Lenarduzzi et al. 2019), which is a curated collection of data coming from 39 Java projects mainly from the Apache Software Foundation ecosystem. Despite belonging to a single ecosystem, the majority of such projects were originally selected by following the diversity guidelines introduced by Nagappan et al. (2013), i.e., they were selected by addressing the representativeness of projects in terms of age, size, and domain, and the Patton’s “criterion sampling” (Patton 2002), namely, they are more than four years old, have more than 200 commits and 100 classes, and have more than 100 issues reported in their issue tracking system. As such, this dataset minimizes by design possible threats to external validity. To further verify the properties of this dataset, we have manually investigated the corresponding Github repositories and discovered that all of them adhere to a strict code of conduct (Tourani et al. 2017) and regularly review source code to improve their quality processes (Pascarella et al. 2018). This analysis further confirmed the suitability of the dataset. It is important to note that nine of the considered systems also appear in the *National Vulnerability Database* (NVD) (2023b), which was initially developed by the U.S. NIST Computer Security Division (2023a) to collect and provide public information about known vulnerabilities affecting software systems and their causes. Such a database includes a comprehensive set of publicly known vulnerabilities: each of them is described through CVE (Common Vulnerabilities and Exposure 2023a) records and is enriched with additional pieces of information such as external references, severity (computed using the Common Vulnerability Scoring System - CVSS), the related weakness type (Common Weak Enumeration - CWE), and the known affected software configurations (Common Platform Enumerations - CPEs). NVD aggregates information from multiple data sources and is widely considered a reliable data source (Alhazmi et al. 2007; Huang et al. 2010; Zhang et al. 2011).

While all the projects were considered when addressing **RQ**_1_ and **RQ**_2_, only the nine systems overlapping with the NVD could be used for **RQ**_3_ as these are the only ones for which we could obtain data on the known vulnerabilities affecting them. Table 1 reports the main characteristics of the projects in our context—we report statistics on their change history with a particular focus on the refactoring operations observed.

**Table 1:**
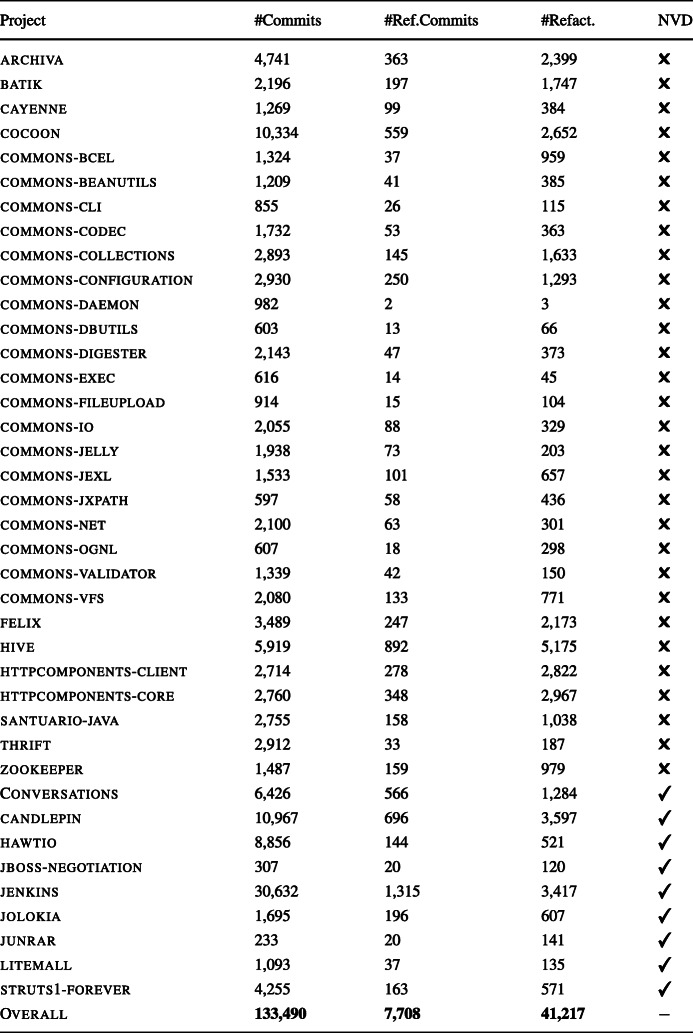
Summary of the considered software projects

The last column ‘NVD’ indicates whether the corresponding project has known vulnerability data

“#Ref.Commits.” refers to the number of commits having refactorings

“#Refact.” refers to the number of refactoring instances observed

### Data Collection

This section describes how we collected each piece of information to address our research questions: developers’ refactoring operations, security metrics, security-related technical debt, and known vulnerabilities that affected the projects considered.

#### Mining Refactoring Data

We mined the entire change history of the considered projects to identify commits where developers applied at least one refactoring. To this aim, we run version 2.2 of RefactoringMiner (Tsantalis et al. 2018) against each source code change. RefactoringMiner is a publicly available tool^5^ that can detect a large number of refactoring types through the analysis of how the Abstract Syntax Tree of a Java class/method has changed with respect to the one of the previous commit. The output of RefactoringMiner is formatted as a JSON file reporting for each commit the set of refactoring operations applied and the classes/methods subject to them. Despite the existence of alternative refactoring detectors (e.g., RefDiff (Silva et al. 2020)), we opted for RefactoringMiner since it is publicly available and has a detection accuracy close to 100%, overcoming the capabilities of other detectors (Tsantalis et al. 2018).

In the context of this study, we selected a set of common refactoring operations having mixed relations with security to uncover possible unexpected and sneaky correlations. Table 2 reports the 12 basic refactoring operations plus two composite refactoring operations (i.e., successive application of two elementary refactorings) we deemed worth investigating. Each row contains a description of how they work and of the possible impact on security. Such expected impacts derive from the refactoring operations’ definition and represent the conjectures we aim to verify in our empirical investigation. In this respect, we formulated the hypotheses we posed for this study and described them graphically in column ‘*H*_*p*_’. An up arrow (‘*↑*’) indicates that we hypothesized a certain refactoring has a positive (good) effect on source code security; a down arrow (‘*↓*’) indicates that we hypothesized a negative (bad) effect. In contrast, the symbol *‘—’* indicates a hypothesis of stability, i.e., the refactoring should not change the security profile of the source code in any way. As further explained in Section 2.5, these hypotheses were instantiated for the three specific research questions. All the selected refactoring operations alter the source code’s internal structure at different granularity levels—ranging from individual attributes to groups of classes.

**Table 2:**
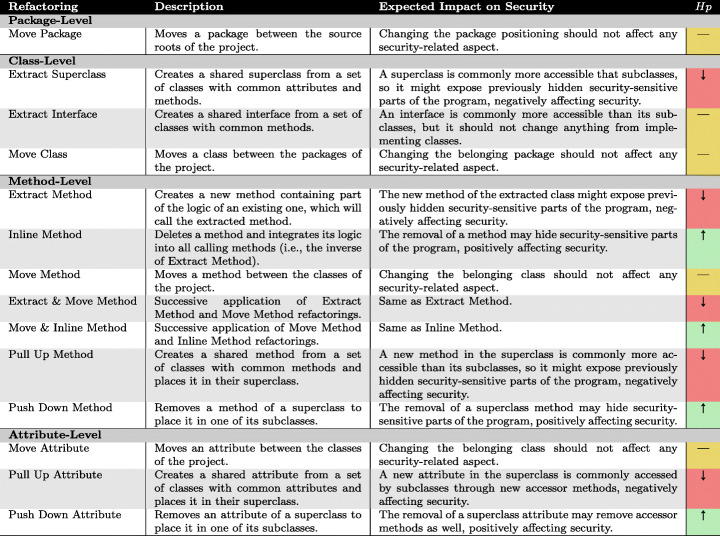
Set of refactoring types selected in this study collected using RefactoringMiner (Tsantalis et al. 2018)

#### Mining Security Metrics

We computed a set of metrics that have been previously used to assess source code security (Abid et al. 2020; Alshammari et al. 2010b; Agrawal and Khan 2014) on all the refactoring commits of the projects. Table 3 reports their names and description. The metrics measure source code against the presence of confidential or sensitive information, e.g., user IDs, authorization tokens, or passwords, that might potentially worsen the security level. For instance, over-exposed (in terms of access specifiers) code fragments might lead to vulnerabilities that can be exploited.

**Table 3:** List of security metrics computed by Surface

Metric	Acronym	Description
**Class-Level**		
Classified Attributes	CA	Number of *classified* attributes of a class, identified through pattern matching heuristics (e.g. password, token).
Classified Methods	CM	Number of *classified* methods of a class, identified through (i) pattern matching heuristics (e.g. validatePassword, generateToken) or (ii) the check of usages of classified attributes.
Classified Instance Variables Accessibility	CIVA	Ratio of non-private and non-static classified attributes out of the total number of classified attributes (CA).
Classified Class Variables Accessibility	CCVA	Ratio of non-private and static classified attributes out of the total number of classified attributes (CA).
Classified Method Accessibility	CMA	Ratio of non-private classified methods out of the total number of classified methods (CM).
Classified Methods Ratio	CMR	Ratio of the number of classified methods (CM) out of all class methods.
Classified Attribute Interactions	CAI	Sum of the number of classified methods that accesseach classified attribute, divided by the product of thenumber of classified attributes and methods (CA × CM).
**Project-Level**		
Critical Classes	CC	Number of *critical classes*, i.e., classes with at least one classified components (classified attribute or method).
Critical Classes Ratio	CCR	Ratio of the number of critical classes (CC) out of all project classes.
Critical Classes Extensibility	CCE	Ratio of non-final critical classes out of the critical classes (CC).
Classified Methods Extensibility	CME	Ratio of non-final critical methods among all classes out of the critical methods among all classes.
Critical Super Classes Ratio	CSCR	Mean of the ratios, for each class, of the number of critical super classes out of all their super classes.
Serializable Critical Classes Ratio	SCCR	Ratio of serializable critical classes out of the critical classes (CC).

To compute these metrics, we developed a tool, which we named Surface, and, for the sake of verifiability, we made it available in our online appendix (Iannone et al. 2022). Our tool is a re-implementation of the one built by Abid et al. (2020), as it was not publicly available. Given a source code file, it first verifies the presence of the security-related keywords identified in Abid et al. (2020) to identify *“classified attributes”* (i.e., class fields that contain confidential or sensitive information), that will be used as a basis for applying further static analyses and computing the other metrics. To this end, we used a set of regular expressions based on those adopted by Abid et al. (2020) to automatically detect all classified code elements (i.e., attributes, methods, and classes). The following box reports the regular expressions used by Surface as a comma-separated list of strings. 
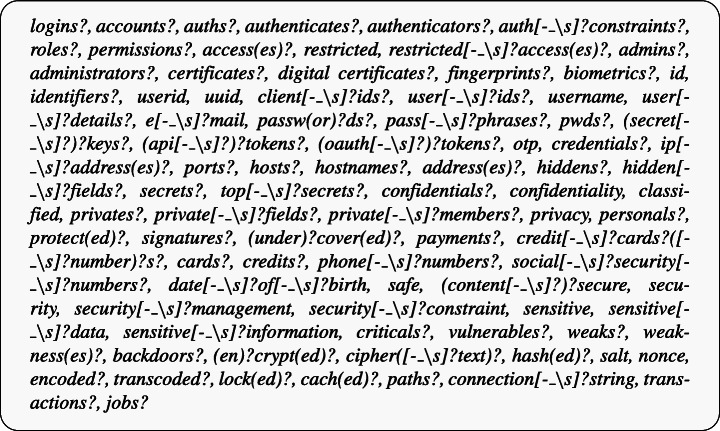


We are aware that most of the metrics are derived from the set of classified attributes; hence they capture similar aspects connected to source code security. Yet, to the best of our knowledge, these are the only ones available that can enable an analysis of the security profile of object-oriented source code in a fully-automated fashion.

To collect the security metrics values, for each commit having at least one refactoring instance, we selected only those Java files directly involved in one of the refactorings made in the commit. Then, we run Surface twice: one time considering the files’ versions before the commit, and one more time on the versions after the commit. The resulting metrics for the previous files’ versions were subtracted from the latest versions, obtaining the *delta* (*Δ*) that represents how much a metric has changed in that commit. It is worth noting that for newly-added files the metrics were left as-is, while for the files deleted in the commit their values resulted to have a negative sign. Afterward, all the class-level security metrics (Table 3) were aggregated to have individual values expressed at the entire commit level. Specifically, the deltas pertaining to the security metrics CA (Classified Attributes) and CM (Classified Methods) were summed together (as they represent counting), while the rest of the metrics were averaged. In this way, we could outline the magnitude of change in the security profile after a commit containing refactorings.

#### Mining Security-related Technical Debt

According to recent findings, SonarQube is among the most popular automated static analysis tools employed in practice (Vassallo et al. 2019; Avgeriou et al. 2021), in addition to being accurate when detecting security violations (Saarimaki et al. 2019). Based on these observations, we selected SonarQube version 7.5 to collect the metrics needed for **RQ**_2_. In particular, we measured the security-related technical debt collecting two kinds of metrics linked to the security profile of applications. On the one hand, for each refactoring commit, we counted the number of violations of security rules (i.e., those belonging to the *“Vulnerability”* group) that SonarQube encountered when analyzing the Java classes of the project’s snapshot after the commit. Such violations indicate that the code is likely to be affected by a software vulnerability, or has laid the foundation for vulnerable code. Moreover, similarly to what we did for **RQ**_1_ (Section 2.3.2), we counted the violations only on those files directly involved in the refactorings that occurred in that commit. On the other hand, we obtained the so-called “security remediation effort”, i.e., a measure of how much effort developers would spend when addressing all the detected violations in that snapshot—based on the violated security rules.

Once we collected all the technical debt-related metrics for all the refactoring commits, we computed the difference (*Δ*) between all the metrics values with their previous version (i.e., the parent commit) to compute the change in the number of violations and remediation effort—analogously to what we did for security metrics in **RQ**_1_. Overall, SonarQube was able to detect 26 different types of security violations. Table 4 reports the top 10 most recurring violations that were found and resolved within the refactoring commits we analyzed. Each rule in the table is accompanied by its description, the number of occurrences, and the corresponding severity level. The full list of rules for is available on the SonarQube website.^6^

**Table 4:** Top 10 most recurring SonarQube rules violated in the selected projects

Security Rule	Description	#	Severity
Class Variable Visibility Check	Class variable fields should not have public accessibility	10,532	Critical
S1313	IP addresses should not be hardcoded	4,328	Critical
S1148	Throwable.printStackTrace(...) should not be called	4,193	Critical
S1444	Public static fields should be constant	3,732	Critical
S2386	Mutable fields should not be public static	1,306	Major
S2755	Fails for DocumentBuilderFactory XXE should be disabled	633	Blocker
S4423	Weak SSL/TLS protocols should not be used	484	Major
S2077	SQL binding mechanisms should be used	426	Major
S2068	Credentials should not be hard-coded	371	Critical
S5542	Encryption algorithms should be used with secure mode and padding scheme	333	Critical

#### Mining Known Vulnerability Data

We only considered vulnerabilities in NVD affecting the considered systems (see Table 1) and specifying the fixing commit (i.e., the one that officially patched a publicly disclosed vulnerability)—otherwise, we could not address our **RQ**_3_, as explained later in Section 2.4. From an operational perspective, we mined the full dump of NVD exploiting CVE-Search project (2023b), allowing the download of a JSON file containing all CVE records updated daily. We obtained the full JSON dump on May 30, 2022. We performed some additional filtering steps to remove incomplete/incorrect data that might have biased our observations: (1) we discarded CVEs that reported commits to different GitHub projects than those considered since we could not establish where the vulnerability was residing; (2) we filtered out vulnerabilities whose fixes were marked as merge commits, as these do not apply any real modification in the project history but simply incorporate the changes (i.e., a set of commits) from a branch into another, i.e., we could not consider them as actual patches since we were interested in getting precise information about the moment when fixes were added into the history rather than the moment when they were sent into the main branch. After this filtering, we ended up with a total of 26 known vulnerabilities of 12 distinct types, pertaining to nine NVD projects. Table 5 reports the 26 vulnerabilities grouped by their vulnerability type (CWE).

**Table 5:** The 26 known vulnerabilities mined from NVD grouped by the 12 vulnerability types

Vulnerability Type	Description	#CVE
CWE-264	Permissions, Privileges, and Access Controls	4
CWE-200	Exposure of Sensitive Information to an Unauthorized Actor	4
CWE-287	Improper Authentication	3
CWE-79	Cross-site Scripting	3
NVD-CWE-noinfo	No sufficient information to classify the vulnerability	2
CWE-254	7PK Security Features	2
CWE-22	Path Traversal	2
CWE-352	Cross-Site Request Forgery (CSRF)	2
CWE-326	Inadequate Encryption Strength	1
CWE-310	Cryptographic Issues	1
CWE-20	Improper Input Validation	1
CWE-835	Infinite Loop	1

### Data Analysis

After collecting the data required to address our research questions, we proceeded with the statistical modeling and the subsequent interpretation.

#### **RQ**_1_ −**RQ**_2_. Refactoring vs. Security-related Metrics and Technical Debt.

The first two research questions aim at understanding the effect of refactoring on indicators of longer-term source code security issues. For both **RQ** s, we employed similar analysis methods.

Starting from the security metrics (Section 2.3.2) and the security-related technical debt (Section 2.3.3) variations observed in the refactoring commits, we converted all *Δ* values into categories that could be better interpreted by humans. If a metric *m* had a *Δ* > 0 in one of the refactoring commits analyzed, the variation was converted into the category *“Increased”*. Similarly, if *m* has a *Δ* < 0, it was converted to the category *“Decreased”*. Otherwise, it was converted to *“Stable”*. It is worth noting that the interpretation of these categories depends on the specific metrics. Let us consider CA (Classified Attributes) metrics as an example. An increased number of classified attributes is generally deemed as something negative, as it indicates an increment in the number of fields holding security-sensitive data. In this case, observing many deltas labeled as *“Increased”* is a negative indication of the security profile of the application.

Afterwards, to address **RQ**_1_ and **RQ**_2_ we built a statistical model for each security metric and technical debt in which we relate the number of distinct refactoring operations applied between *c*_*r*− 1_ and *c*_*r*_ as well as other control variables to the three categories mentioned above, i.e., *“Increased”*, *“Stable”*, and *“Decreased”*. Approaching the research questions in this manner allowed us to verify which refactoring types have connections to security indicators and whether the effect of those refactoring operations is positive or negative.

More specifically, we considered the categorical values associated with each refactoring commit as *dependent variables*. The number of refactoring operations for each of the 14 considered types were treated as our *independent variables* in all the models. Furthermore, we computed three additional metrics that acted as the *confounding variables*, namely the factors that might significantly influence a dependent variable regardless of the values of the independent variables (Kutner et al. 2005). They are:

1. The number of lines of code (LOC) of the files’ versions that underwent to refactoring, i.e., immediately before the commit detected by RefactoringMiner. All the LOC values were averaged to have a single summarized value for an entire commit. This metric has often been associated with a reduction of source code quality, and dependability (El Emam et al. 2001; Koru and Liu 2005; Zhang 2009). The inclusion of this confounding factor was motivated by the assumption that working on files with many lines of code might have a higher chance of increasing the values of security indicators or contributing to the introduction of vulnerabilities with respect to smaller files.
2. The Weighted Methods per Class (WMC) (Chidamber and Kemerer 1994) computed on the files’ versions that underwent refactoring, i.e., immediately before the commit detected by RefactoringMiner. All the WMC values were averaged to have a single summarized value for an entire commit. This metric represents the sum of McCabe’s cyclomatic complexity (McCabe 1976) values computed on the class’s methods. In this case, the negative impact of code complexity on vulnerabilities has been previously assessed (Chowdhury and Zulkernine 2011; Shin and Williams 2008).
3. The code churn, i.e., the amount of code added/deleted in the commit that touched the files’ that underwent to refactoring. All the churn values were summed to have a single summarized value for an entire commit. Previous work has shown that the higher the churn of two subsequent commits, the higher the likelihood to introduce issues in the code (Nagappan and Ball 2005). The negative impact of churn metrics has also been assessed when considering software vulnerabilities (Shin et al. 2010).

Such metrics were extracted using PyDriller (Spadini et al. 2018), which allows straightforward analyses of projects’ change history, and Lizard,^7^ which parses the source code and automatically extracts a set of common structural metrics from the source code.

Furthermore, we encoded the projects as 39 different binary variables to capture any possible random effect coming from a specific project.

Having a categorical dependent variable, we fit a mixed-effect Multinomial Log-Linear model (Theil 1969), a classification method that can generalize logistic regression to multiclass problems, so fitting our case. The models were built using the R toolkit exploiting the multinom model of the package nnet.^8^

The choice of a mixed-effect Multinomial Log-Linear model was driven by multiple observations. First and foremost, it fits the multiclass problem we intended to model when building a theory of how refactoring is related to security. Second, it outputs precious pieces of information that can be used to interpret the results, as detailed in the remainder of the section. It indeed provides statistical codes through which each individual refactoring type can be assessed against its statistical relevance for the problem under analysis—as such, we could identify the refactoring types having a statistically significant connection to security. Furthermore, it returns the *odds ratios* (OR) (Bland and Altman 2000)—i.e., the exponential of the model’s coefficients—that provide a measure of the actual impact of the associated variables, i.e., the refactoring type. Such interpretation complements the statistical codes, providing a more practical measure to interpret the effects of refactoring on security. Other research methods, e.g., correlation analysis, cannot provide such a comprehensive and tangible assessment of our hypotheses. Perhaps more importantly, it is important to remark that security might and might not be affected by the refactoring; other factors might play a role. The statistical modeling exercise allowed us to specify a set of confounding variables and, for this reason, assess the impact of refactoring while keeping other factors into account.

When building the models, we took the problem of multicollinearity into account. This arises in cases where two or more independent variables are linearly correlated, and one can be predicted from the other, possibly biasing the model’s fitting capabilities and how the results are interpreted. In this respect, we first verified the normality of the distributions of the independent variables employing the Anderson-Darling normality test (Anderson and Darling 1952). Such a test verifies whether a given sample follows a theoretical distribution, i.e., the normal one. For each independent variable, we compared its distribution with a normal distribution having the same mean and standard deviation of the sample. As a result, all the test runs failed to reject the null hypothesis, hence indicating that our data are not normally distributed. Because of the non-normality of any of the independent variables samples, we computed the Spearman’s rank correlation (Spearman 1961) between all possible pairs of independent variables to determine whether there are strongly correlated pairs (i.e., variables for which the Spearman’s *ρ* > 0.8). This step did not eventually find any correlated variables, meaning that the independent variables’ distribution was different enough to be used together in the statistical models.

As for the interpretation of the results, it is worth noting that the model’s logit coefficients *c*_*i*_ are relative to a reference category and indicate how the independent variables vary the chances of the dependent variable being affected with respect to the reference category. We set such a category to *“Stable”* to estimate how the various independent variables, i.e., the refactoring operations, likely change in *either* a positive or negative direction the stability of security indicators. For instance, if we have the refactoring type *r*_*i*_ that presents a logit coefficient *c*_*i*_ = − 1.50 in the model built when analyzing the decrease in the security metric *s*_*j*_, this means that a one-unit increase of *r*_*i*_ would lead to an increase of the chances of *s*_*j*_ to remain stable.

After obtaining the logit coefficients, we computed the odds ratios (ORs) using the exponential function ($e^{c_{i}}$). In our case, the ORs complement the interpretation of the results: for an independent variable, i.e., a refactoring type, it indicates the increment of chances for a class to increase/decrease the value of a security metric (**RQ**_1_) or a technical debt metric (**RQ**_2_) as a consequence of a one-unit increase of the refactoring. With the OR values, we could quantify the extent to which the application of refactoring impacts security metrics and debt, hence giving a more practical sense to the coefficients obtained when running the models.

#### **RQ**_3_. Refactoring vs. Known Vulnerabilities

The last research question measures the extent to which refactoring operations contribute to the introduction of vulnerabilities. To address it, the first challenge was concerned with mining the commits responsible for the introduction of vulnerabilities.

To obtain these *vulnerability-contributing commits* (a.k.a. VCCs) (Meneely et al. 2013), we have followed the idea behind the well-known SZZ algorithm (Sliwerski et al. 2005), which recovers the set of commits that likely introduced a defect starting from a bug-fixing commit using the git-blame functionality on the lines deleted during the fix. Despite SZZ has been envisioned to fetch the commits that induce traditional defects (Rodríguez-Pérez et al. 2018b), it has also been exploited to fetch vulnerability-contributing commits (Perl et al. 2015; Yang et al. 2017; Iannone et al. 2022). Yet, it has been subject to adjustments and improvements. In this work, we adopted a set of heuristics to reduce the amount of noisy results. Specifically, for each Java file *F*_*i*_ modified in a vulnerability-fixing commit *f*, we run the git-diff functionality to obtain the list of added and deleted lines in *F*_*i*_ and then applied two strategies to obtain the VCCs. Firstly, we run the git-blame command to obtain the commits that last changed the lines deleted in *f*. Secondly, we blamed the lines “around” continuous blocks of changes—generally representing new checks—made only of added lines. The former was done to recover those commits that have likely added flawed pieces of code—e.g., a call to an improper input validation function or the use of an obsolete cryptography algorithm. Instead, the latter can reach the commits touching the code areas that lacked solid control mechanisms. The only exception was made for blocks made of totally new functions or methods, as they can be placed anywhere, rendering their contextual lines irrelevant. In addition, we did not blame empty and comment lines, and irrelevant non-source code files—e.g., documentation, build, blob, and test files—as they do not generally contribute to a vulnerability. What is more, we did not consider the VCCs that merged changes from multiple commits, as they do not report real modifications per se. It is worth noting that vulnerabilities could have been fixed by multiple fixing commits; in such cases, we united the set of VCCS obtained from each fixing commit to build the final set. The described procedure was implemented exploiting PyDriller (Spadini et al. 2018) repository mining library with the help of the parsing library Lizard^9^ to apply our heuristics.

Once we had detected the vulnerability-contributing commits, we could verify in how many cases such commits were also marked as refactoring commits (collected as described in Section 2.3.1). Therefore, we sought to elicit the amount of vulnerability-contributing commits for which refactoring might have played a role. When addressing **RQ**_3_, we also reported the results by considering each refactoring type individually, hence assessing if a particular operation is more likely to contribute to the introduction of a vulnerability.

### Hypotheses and Statistical Verification

Once we completed the statistical modeling, we proceeded with the verification of the high-level hypotheses formulated in Table 2. More specifically, we first refined them to derive more concrete null and alternative hypotheses to test the research questions in this study. In the cases of *Move Package*, *Extract Interface*, *Move Class*, *Move Method*, and *Move Attribute* refactoring, we defined the following null hypothesis:

Hn_1_: The refactoring has a *significant impact*, either positive or negative, on security properties.

Our alternative hypothesis was, instead:

Ha_1_: There is *no significant impact* of the refactoring on security properties.

In the context of **RQ**_1_ and **RQ**_2_, we rejected the null hypotheses if the coefficients of the statistical models built to understand the *increase* and *decrease* of the security properties were not significant or negative. In the latter case, the coefficients of the Multinomial Log-Linear model would indicate that refactoring operations tend to increase the likelihood of security metrics/debt being stable, hence rejecting the null hypothesis in favor of the alternative one. As for **RQ**_3_, we run the non-parametric Mann-Whitney U test (Mann and Whitney 1947) (with *α* =0.5) on the distribution of refactoring operations within VCCs and non-VCCs commits. We rejected the null hypothesis if *α* > 0.05. We also measured the effect size of the differences identified in the two distributions using Cohen’s *d* (Cohen 2013). We followed well-established thresholds for interpretation: 0.2 for *Small*, 0.5 for *Medium* and 0.8 for *Large* effect size (Cohen 2013).

With respect to *Extract Superclass*, *Extract Method*, *Extract & Move Method*, *Pull Up Method*, and *Pull Up Attribute* refactoring, our null hypothesis was:

Hn_2_: There is *no significant impact* of the refactoring on security properties.

The alternative hypothesis in this case was:

Ha_2_: The refactoring has a *significant negative impact* on security properties.

In **RQ**_1_ and **RQ**_2_, we rejected the null hypothesis if we observed (i) both significant coefficients and (ii) positive coefficients in the statistical model built to understand the *increase* of security metric/debt values and negative coefficients in the statistical model built to understand the *decrease* of security metric/debt values. In the latter case, indeed, the statistical model coefficients would tell us that the application of refactoring operations tends to increase the likelihood of the metrics/debt being increased, hence indicating their deterioration. As for **RQ**_3_, we still relied on the same outcomes and interpretation of the Mann-Whitney U test (Mann and Whitney 1947) and Cohen’s *d* (Cohen 2013).

Finally, when it comes to *Inline Method*, *Move & Inline Method*, *Push Down Method*, and *Push Down Attribute* refactoring, the null hypothesis was set to:

Hn_3_: There is *no significant impact* of the refactoring on security properties.

The alternative hypothesis in this case was:

Ha_3_: The refactoring has a *significant positive impact* on security properties.

As for **RQ**_1_ and **RQ**_2_, we rejected the null hypothesis if we observed (i) both significant coefficients and (ii) positive coefficients in the statistical model built to understand the *decrease* of security metric/debt values and negative coefficients in the statistical model built to understand the *increase* of security metric/debt values. In the latter case, the statistical coefficients would indicate that the application of refactoring operations has the tendency to increase the likelihood of the metrics/debt being decreased, which would mean that the security profile would improve. In **RQ**_3_, we relied on the outcomes and interpretation of the Mann-Whitney U test (Mann and Whitney 1947) and Cohen’s *d* (Cohen 2013).

### Verifiability and Replicability

In order to allow our study to be verified and replicated, we have published the complete raw data, along with the data collection and analysis scripts in our online appendix (Iannone et al. 2022). The ‘README.md' file contains more precise instructions on how to use our artifacts to replicate the study.

## Analysis of the Results

In this section, we report the results of the empirical study, discussing them by research question.

### **RQ**_1_. To What Extent Do Refactoring Operations Impact Security Metrics?

In **RQ**_1_, we sought to understand the relation between refactoring and security metrics. Table 6 reports for each refactoring type the sign of the logit coefficients (within a circle) and the value of the ORs obtained for the Multinomial Log-Linear models built to understand the decrease and increase of the security metrics considered in the study. The coefficients of the variables that turned out to be statistically significant are reported with a colored symbol (green for , red for ), otherwise are left white. In addition, the cells with a gray shade indicate that the impact of that type of refactoring rejects the null hypothesis formulated in Section 2.5—i.e., the impact turned out to be in line with our expectations. For the sake of readability, we did not report the coefficient signs and ORs values of the confounding factors (LOC, WMC, and code churn) considered when building the models, but we discuss their role in our analysis and report the full results in our online appendix (Iannone et al. 2022). Looking at the table, we can immediately observe that the refactoring types *Move Method*, *Move Attribute*, *Extract Superclass*, and, to a lesser extent, *Push Down Method*, always had positive coefficients in all the 13 models built for the 13 security metrics; this means that moving code components (i.e., attributes or methods) from a class to another or optimizing the degree of code reuse (i.e., creating better class hierarchies) have the effect of varying the security profile of an application, either positively or negatively. Moreover, the *Extract Superclass* refactoring had a particularly strong impact on CCR and CSCR metric, i.e., their ORs are the largest among all the other models. As such, extracting new classes in hierarchies impacts (i) the ratio of the number of critical classes to the number of classes in the entire project (ii) and the number of superclasses in all the class hierarchies (CSCR), respectively. Although these findings suggest that these refactoring types have a random effect on security metrics, it is reasonable to believe that the impact is determined by their specific application, i.e., the security profile is affected differently depending on how developers apply the refactoring operations.

**Table 6:**
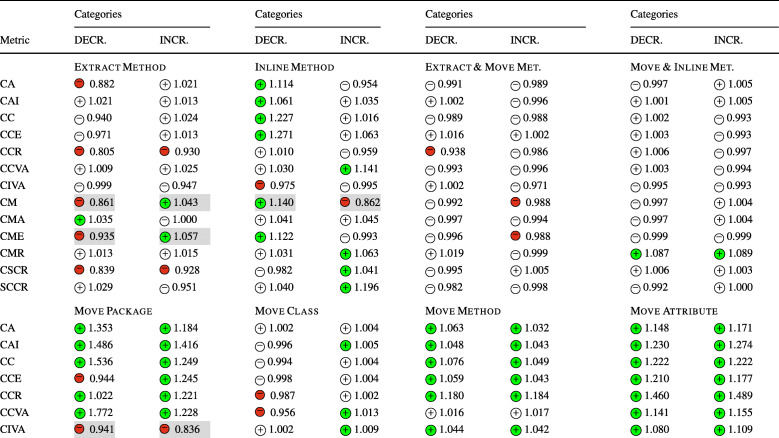

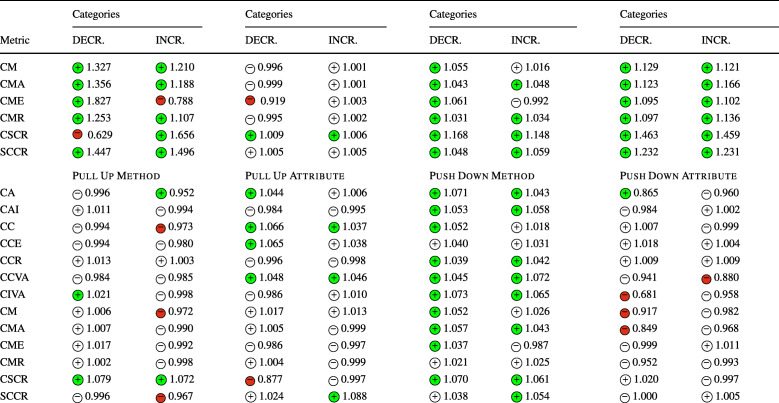

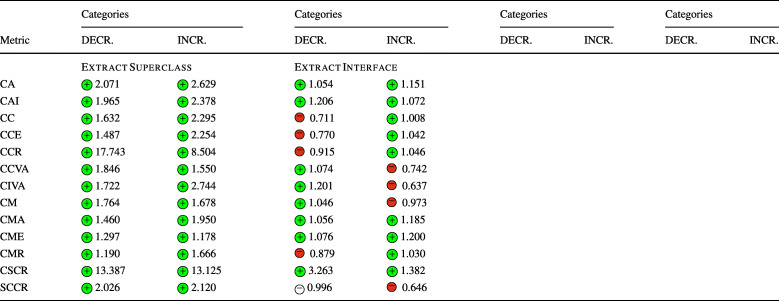
The impact of each refactoring type on security-related metrics (**RQ**_**1**_) represented via the sign of the models’ coefficients (colored if *p* < 0.05) and their odds ratios

The category ‘DECR.’ represents the cases where *Δ* < 0, while ‘INCR.’ represents *Δ* > 0.The cells in gray indicate the acceptance of the related alternative hypotheses (Ha_1_) formulated for **RQ**_**1**_. (Section 2.5)

Surprisingly, refactoring types for which we expected no impact, such as *Extract Interface* and *Move Package*, still exhibited a statistically significant mixed effect on the security metrics. Between the two, *Extract Interface* showed a clearer behavior. On the one hand, it tends to increase the value of CC, CCE, CCR, and CMR metrics—i.e., increasing the number of critical classes. On the other hand, it keeps reducing CCVA, CIVA, and CM metrics—i.e., reducing both the accessibility of instance and class variables, and the number of classified methods. This means that re-organizing the classes’ interfaces helps keep the number of critical attributes and methods under control, still increasing the risk of introducing too many critical classes. Differently, only for the CIVA metric, the *Move Package* refactoring matched our expectations: moving classes among packages does not affect this metric at all. This might be explained by the fact that commits applying package restructuring are generally not done in a fully-isolated manner but are applied in the context of other changes—which have a mixed impact.

*Extract Method*, *Inline Method*, and *Move Class* still exhibited mixed effects on the various security metrics, but with much lesser significance than other refactoring types. The only cases where the null hypotheses were rejected were for *Extract Method* for CM and CME metrics, and also for *Inline Method* for CM metric. This is quite straightforward to comprehend. *Extract Method* creates new methods from a piece of code in existing methods, likely introducing new classified methods if the extracted logic deals with classified attributes. At the same time, *Inline Method* refactoring eliminates redundant methods, with a high chance of removing classified methods and reducing access to classified attributes. This was the case of project Conversation at the revision 14cfb609. In such a commit, the utility class CryptoHelper was streamlined into three new classes, all placed in package crypto/sasl. Additionally, the XmppConnection class—in change or managing XMPP connections—was refactored to inline the two methods sendSaslAuthPlain() and sendSaslAuthDigestMd5() into processStreamFeatures(). Indeed, the two removed methods were only called by processStreamFeatures(), so driving the developer to apply two *Inline Method* refactorings—the commit message confirms this intentions, i.e., *‘Refactor authentication code’*. In this respect, sendSaslAuthPlain() was a classified method, as it access to security-sensitive data, i.e., account instance variable in this case. Hence, its removal led to the reduction of one from the CM metric. This variation translates into a reduction of the overall application’s attack surface. Indeed, the metrics proposed by Alshammari et al. (2012) penalize those classes exposing too many methods that access classified components, as attackers might leverage them to carry out attacks. This explains why *Inline Method* refactorings have been seen as beneficial from this perspective is beneficial. Such an example also opens an interesting observation. While a securely-designed class should minimize the number of methods with the responsibility of accessing security-critical data, good design practices for maintainable code recommend creating many small and cohesive methods. Thus, creating both maintainable and secure code demands particular care not to create too many classified methods but still avoiding making poorly cohesive and long methods. In other words, accessing security-critical data should be reserved for only an essential set of elected classes and methods. Similarly, in commit 43531113 of the same project, an *Extract Method* refactoring was applied on sendBindRequest() to extract sendIqPacket() method, which happened to access security-sensitive data—so, it was branded as a new classified method. While this method is not necessarily a security issue, its presence increases the application’s attack surface, giving attackers an additional method to leverage for its purpose.

All the other refactoring types, i.e., *Move Class*, *Pull Up Attribute*, *Push Down Attribute*, and *Pull Up Method*, appears to have limited or no impact on any security metrics. Perhaps more interestingly, the composite refactoring types considered in the study, namely *Extract & Move Method* and *Move & Inline Method*, seem to show a “mixture” of the behaviors of their individual operations. This had the curious effect of having almost no statistical significance for all 13 models. Yet, this could also be caused by the limited amount of composite refactorings observed in our dataset.

When considering the impact of the confounding factors, i.e., LOC, WMC, and code churn, we could notice that only code churn has a statistically significant relationship with all dependent variables. On the contrary, WMC turned out to have a poor impact on the security metrics, indicating that the complexity of methods is not related to the number of critical components in the source code. All in all, the ORs for all confounding factors are still very low, translating into a very weak effect on security.

Last but not least, we also found that in some cases, the projects themselves turned out to be significant for the explanation of the dependent variable. From a practical point of view, this means that the peculiarities of the projects have some influence on the changes to the global security profile. Our study cannot uncover the reasons behind this finding, as it would deserve further investigation. Yet, it is reasonable to believe that project-specific properties exist, e.g., contribution guidelines (Elazhary et al. 2019), code of conducts (Tourani et al. 2017), and more, that make developers more or less prone to introduce vulnerabilities. 
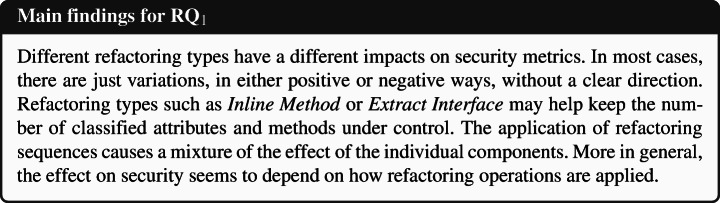


### **RQ**_2_. To What Extent Do Refactoring Operations Impact Security-Related Technical Debt?

In **RQ**_2_, we investigated the relation between refactoring and security-related technical debt, measured through the number of security violations detected by SonarQube and the security remediation effort. Similarly to **RQ**_1_, Table 7 reports the sign of the logit coefficients (within a circle) and the value of the ORs obtained for the Multinomial Log-Linear models for each refactoring type. Here too, the coefficients of the variables that turned out to be statistically significant are reported with a green or red , otherwise are left white. Whenever the impact of a refactoring type rejects a null hypothesis formulated in Section 2.5 the related cells are depicted in gray. The table does not report the confounding factors (LOC, WCM, and code churn), but these are reported in the raw results in our online appendix (Iannone et al. 2022).

**Table 7:**
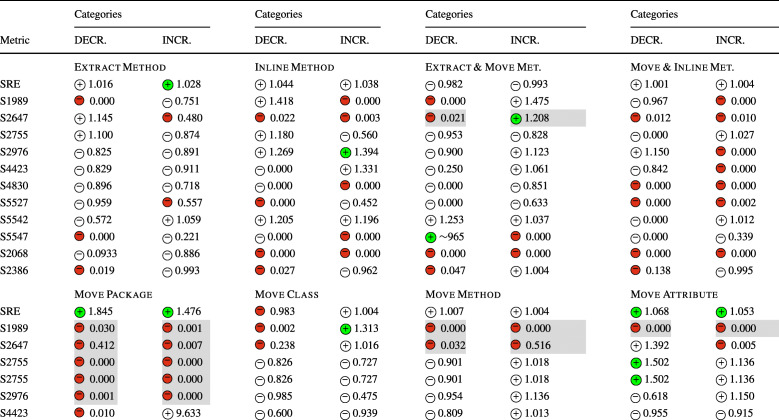

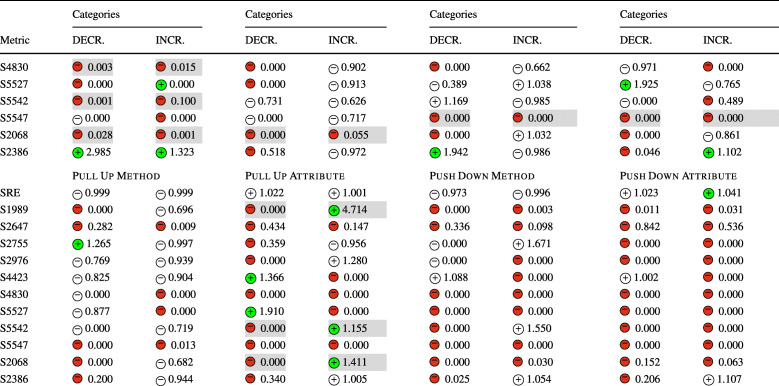

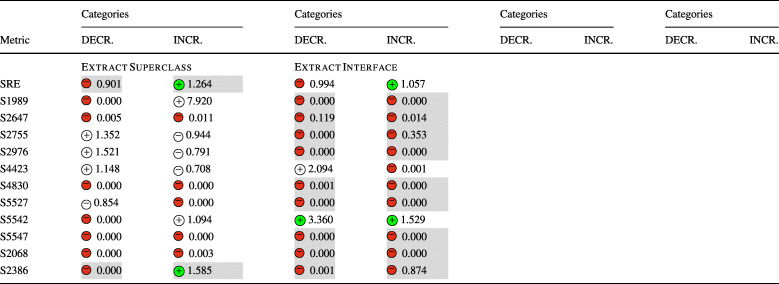
The impact of each refactoring type on security debt and violations (**RQ**_2_) represented via the sign of the models’ coefficients (colored if *p* < 0.05) and their odds ratios

The category ‘DECR.’ represents the cases where *Δ* < 0, while ‘INCR.’ represents *Δ* > 0. The cells in gray indicate the acceptance of the related alternative hypotheses (Ha_2_) formulated for **RQ**_2_. (Section 2.5)

SRE stands for “Security Remediation Effort”, measuring the security technical debt

Looking at the results, we could immediately notice the predominant presence of negative coefficients associated with both the decrease or increase of rule violations, implying that the majority of refactoring operations tend to keep the number of violations stable. This is particularly evident for *Extract Interface*, *Move Package*, *Push Down Method*, and *Push Down Attribute*. In other words, these refactoring types generally do not introduce or resolve any security-related violation. This is in line with their definition. *Extract Interface* and *Move Package* do not overhaul the code structure of the involved classes, so it is reasonable that they do not affect any security rule. The refactoring operations that push attributes or methods down to class hierarchies—i.e., *Push Down Method*, *Push Down Attribute*—do not seem to affect security rules in any way. However, both *Move Package* and *Move Attribute* are connected to a variation of the security remediation effort. This could be explained by the fact that these kinds of changes are made in conjunction with other changes that introduce security-related technical debt.

**Fig. 3:**
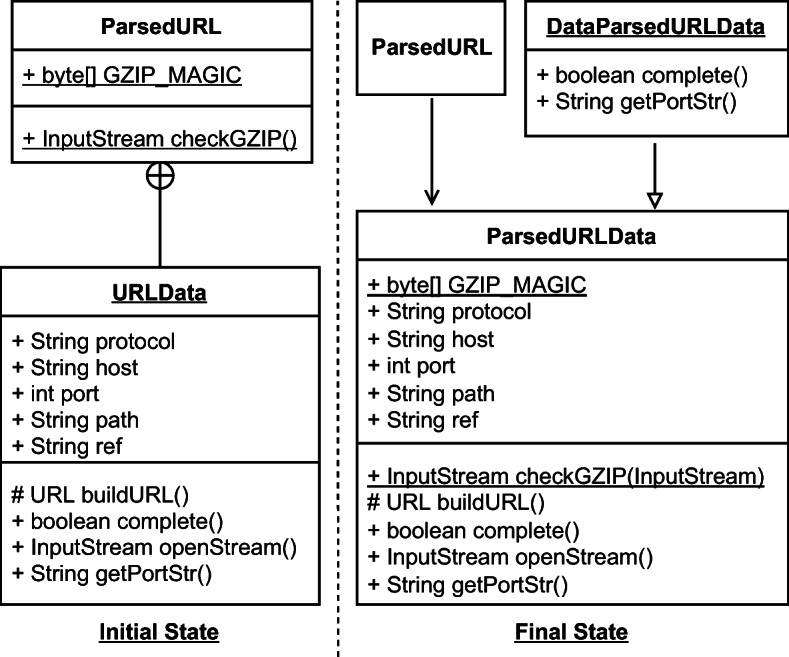
Graphical representation of the *Extract Superclass* refactoring applied in revision e28370d2 in project batik

Despite these results, there are some notable exceptions worth analyzing. *Extract Superclass* refactoring significantly increases the chance of increasing the security remediation effort. Such a refactoring type also tends to violate rule S2386, i.e., *‘Mutable fields should not be* public static’. Violating such a rule has a security implication, as the presence of public static fields expose mutable objects or arrays to changes by malicious users. Such bad practices are also categorized by CWE-582: *‘Array Declared Public, Final, and Static’*, CWE-607: *‘Public Static Final Field References Mutable Object’*, and CWE-766: *‘Critical Data Element Declared Public’*, all representing weaknesses in the source code that attackers can leverage to carry out attacks. In other words, mutable objects should not be leaked to client programs as they can violate class invariants and disrupt the normal execution flow of the target application—if they have access to its runtime. Moreover, violations of this rule also imply a degradation of encapsulation, further motivating the importance of resolving it early. Curiously, an increase in security remediation effort also happens with *Extract Interface* refactoring. Despite both refactoring operations aiming to simplify the hierarchical organization of a project, they should be made with caution as they have a high risk of increasing the remediation effort. Let us consider an example in project batik at the revision e28370d2, also shown in Fig. 3. The commit applied a series of refactorings to reorganize some hierarchical structures in the util package—as also stated in one paragraph of the full commit description: *‘[...] Cleaned up, made easier to extend and pulled several inner class out of* ParsedURL [...]’. In particular, the class URLData was first promoted from a static nested class to a first-level public class, renamed to ParsedURLData and then reorganized to have a new subclass called DataParsedURLData—hence, an *Extract Superclass* was applied. In the end, the ParsedURL class was streamlined to favor the new extension. In applying these changes, the former nested class had five public attributes left unchanged when the class was made public. This caused SonarQube to recognize a violation to rule S2386 for the five attributes, as now they can be freely modified by an external client program. In this case, we observe that the refactoring alone is not the direct cause of the violation, but the way it was applied led to the creation of extra public static fields. To conclude, *Extract Superclass* and *Extract Interface* refactorings should be applied with particular care as they have also been seen to disrupt all the security metrics (**RQ**_**1**_).

Another exception occurs with *Extract & Move Method* refactoring, that (1) negatively impacts rule S2647, i.e., *‘Basic authentication should not be used’* and (2) increases the chances of rule S5547, i.e., *‘Cipher algorithms should be robust’* being removed—as it can be observed from its high OR. The individual refactoring operations, i.e., *Extract Method* and *Move Method*, do not appear to be connected with these rules in any form, while their combination has observable effects. Analyzing this case further, we could not identify specific reasons why the combination of multiple refactorings has a higher impact than individual refactoring types, yet we can suppose that our results represent a reflection of the number of changes applied, i.e., more changes affect security more than individual ones. Nonetheless, the effect of refactoring sequences is something that might be worth further analyzing in future work.

As in **RQ**_1_, it is worth noting that the results were achieved while controlling for several confounding factors. Similarly to the previous discussion, the confounding factors are generally not statistically significant in any model, i.e., they are not correlated with the increase or decrease of security-related technical debt. Likewise, the projects turned out to be significant, somehow confirming that there exist some project-specific attributes that might influence the security technical debt. 
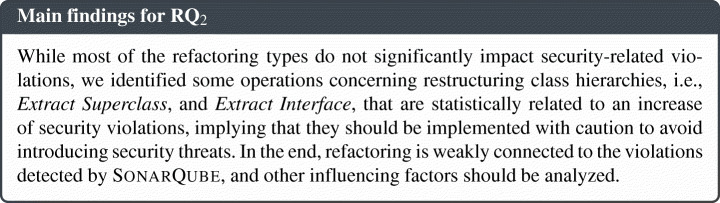


### RQ_3_. To What Extent Do Refactoring Commits Contribute to the Introduction of Real Software Vulnerabilities?

Our third research question investigated the relationship between refactoring and the introduction of known vulnerabilities reported in the National Vulnerability Database (NVD).

It is worth recalling that for this research question, we focused on nine of the projects considered in the study (see Table 1). This subset of projects is affected by 26 known vulnerabilities, i.e., 26 different CVE records, whereas the number of distinct VCCs was 103—there were some cases of commits contributing to more than one vulnerability. Table 8 reports the descriptive statistics of the distribution of refactoring operations, grouped by type, in such VCCs. In the first place, our results showed that the number of VCCs with at least one refactoring was 34, i.e., 33.01% of the VCCs contained at least one instance of a refactoring operation. While this seems to indicate that refactoring might have a connection with the introduction of vulnerabilities, a closer look indicates a lack of causal relationship between the refactoring activities performed by developers and the introduction of vulnerabilities, i.e., the fact that refactoring is performed does not imply that it is the root cause of the vulnerability introduction.

**Table 8:** The main descriptive statistics pertaining to the unique VCCs of the nine projects appearing in NVD. *N* =103

Refactoring	Total	Min	Med	Max	Mean	Std. Dev.
Package-Level						
Move Package	0	0	0	0	0.000	0.000
Class-Level						
Extract Superclass	5	0	0	1	0.049	0.216
Extract Interface	1	0	0	1	0.010	0.099
Move Class	7	0	0	3	0.068	0.377
Method-Level						
Extract Method	24	0	0	6	0.233	0.819
Inline Method	3	0	0	2	0.029	0.220
Move Method	53	0	0	40	0.515	3.983
Extract & Move Method	14	0	0	3	0.136	0.465
Move & Inline Method	2	0	0	1	0.019	0.139
Pull Up Method	251	0	0	231	2.437	22.785
Push Down Method	2	0	0	1	0.019	0.139
Attribute-Level						
Move Attribute	17	0	0	14	0.165	1.387
Pull Up Attribute	126	0	0	81	1.223	9.045
Push Down Attribute	0	0	0	0	0.000	0.000

A more in-depth analysis reveals that the refactoring types that occurred the most in the VCCs were *Pull Up Method* and *Pull Up Attribute* with 251 and 126 instances, respectively. Both refactoring types deal with generalization, hinting that complex restructuring activity (i.e., modifying hierarchies) are often present when vulnerabilities are introduced—this is partially in line with the results observed in the context of **RQ**_2_.

Nonetheless, we also observed their very high standard deviation values that, combined with much lower mean values, imply that the distribution of these refactoring types across the commits is “irregular”—i.e., there are commits with a considerable number of *Pull Up Method* and *Pull Up Attribute* and commits without any of them. For instance, the Jenkins’s commit 70c10658 has over 200 instances of *Pull Up Method* and over 80 of *Pull Up Attribute*. Such a commit touches over 300 different files and represents a crucial commit for the project as it marks the moment when Jenkins was forked from Hudson (its original project). This suggests that some projects, like Jenkins, are characterized by large and poorly cohesive commits, which have higher chances to touch critical parts of the code, possibly introducing defects and security flaws. Hence, a “chaotic” development process might be the actual reason behind the introduction of vulnerabilities—as part of our future research agenda on the matter, we plan to investigate this aspect further.

From a different point of view, the nine NVD projects had a total of 7,708 refactoring commits (i.e., commits with at least one refactoring instance), 34 of which contributed to the introduction of a vulnerability, accounting for 0.44% of the total. This further supports the fact that refactoring alone is not the main responsible for the introduction of vulnerabilities, but rather a co-occurring phenomenon that, in some cases, might worsen the situation, especially when touching several components.

To assess whether the number of a specific refactoring type was statistically significant, we run a one-tailed Mann-Whitney U test (Mann and Whitney 1947) for each refactoring type on both the sets (i.e., the refactoring commits contributing to vulnerabilities versus those that did not contribute), for a total of 14 test runs. We discovered that the distribution of *Extract Superclass* and *Extract & Move Method* instances for VCCs is significantly higher than the distribution for non-VCCs. (*p* < 0.05). At the same time, Cohen’s *d* (Cohen 2013) is lower than 0.2, indicating a very small effect size. In other words, *Extract Superclass* and *Extract & Move Method* occurs more often in VCCs, but still in a limited way. *Extract Method* and *Move Method* refactorings do not appear to show any connection with VCCs, hence suggesting that basic refactorings touching few code components are less likely to contribute to the emergence of a vulnerability. On the contrary, only for *Pull Up Method* and *Pull Up Attribute* the effect size appeared large (*d* > 1.2), but without any statistically significant difference highlighted by the Mann-Whitney U test. The full results of such tests are reported in our online appendix (Iannone et al. 2022).

**Fig. 4:**
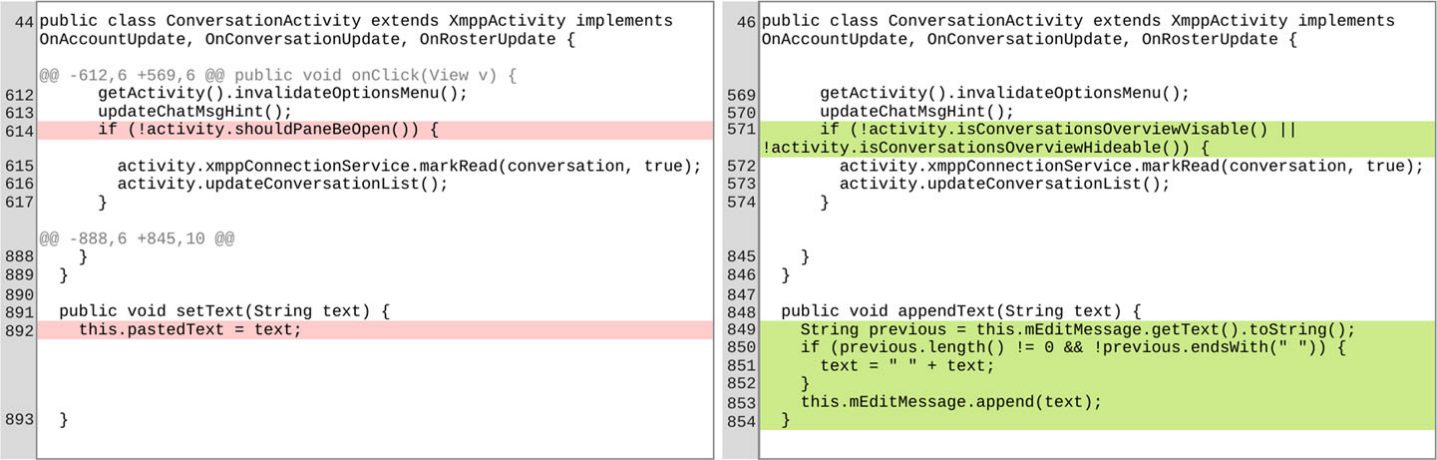
Part of the diff of the commit e45d7bda of Conversations. The focus is given on the root cause behind the vulnerability CVE-2018-18467, i.e. caused by the addition of an incomplete appendText() method during the refactoring


Going more in-depth, let us consider the example reported in Fig. 4. It shows the diff of the commit e45d7bda of the project Conversations, an XMPP client for Android that allows the creation of private chats with other users. The commit message states that a *“UI code refactoring”* was applied. The modification impacted three different files. Four years later, the modification resulted in being one of the causes that led to vulnerability CVE-2018-18467, which allowed an attacker to append a custom text to an existing conversation (with a draft message) by sending an intent from another application. While the commit message suggests code refactoring as the main activity performed, the vulnerability was not due to the refactoring itself, but rather to the addition of the appendText() method in the class ConversationFragment. This allowed appending any text to an existing conversation without adequately checking if an external application was trying to append a text content to an existing draft message through an Intent, leading to the vulnerability described in CVE-2018-18467. In other words, the vulnerability was involuntarily introduced while the committing author was doing some refactoring and code clean-up. In the example, RefactoringMiner only managed to mine two instances of *Inline Method*, which only represent a small part of the total modifications made. Thus, we can conclude that refactoring is often not the direct cause of vulnerabilities but rather a co-occurring phenomenon.

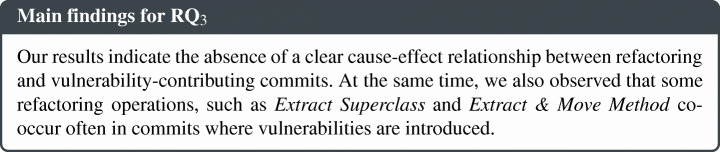


## Discussion and Implications

This section further discusses the main results achieved in our study and reports their implications for researchers and practitioners.

### Discussion: Connecting the Dots

The results of our three research questions allowed us to quantify the role of refactoring on three critical aspects of software security, such as its impact on security metrics, technical debt, and introduction of known vulnerabilities. Moreover, the statistical analyses conducted enable a more general and conclusive discussion of the initial hypotheses formulated in Table 2.

By summing all up, we can provide three main insights. First of all, when looking at the big picture, we can conclude that refactoring has only a *limited effect* on software security. Most of the refactoring operations considered in the study do not lead any security indicators to vary consistently and/or significantly.

As we learned from **RQ**_1_, it is indeed possible that some refactoring operations may influence security metrics depending on how they are applied, while they rarely have an impact on technical debt (**RQ**_2_) and introduction of known vulnerabilities (**RQ**_3_); similarly, it may happen that a change accompanied by refactoring can contribute to a vulnerability without affecting any security metrics or increasing the technical debt value. This is the case of the example shown in Fig. 4 in which neither Surface nor SonarQube detected any difference between the commit containing the refactoring (e45d7bda) and its predecessor (i.e., the *Δ* was mapped to the “Stable” category). Hence, these results partially contradict the preliminary findings reported by Abid et al. (2020): when studying the matter on a larger scale, it comes out that most refactoring operations do not directly impact the security of software systems but are rather co-occurring phenomena.

However, some exceptions have been observed, especially when considering security technical debt. According to our results, the *Extract Interface* refactoring provides a significant increase in security-related technical debt but might have positive effects on some security metrics, e.g., reducing the number of classified methods (CM metric). This result supports and further stimulates the research efforts on the construction of automated refactoring recommenders that might balance quality improvements and security threats, as initiated by Abid et al. (2020).

To broaden the scope of the discussion, our overall findings do not match with the results previously obtained when studying the relation between refactoring and defects (Bavota et al. 2012; Di Penta et al. 2020). In particular, this is the case of the refactoring types dealing with the generalization: while Bavota et al. (2012) and Di Penta et al. (2020) found these operations to be sometimes defect-inducing, we discovered that they can instead provide some benefits to security aspects connected to attribute encapsulation. We see two main points here. On the one hand, these differences corroborate the conclusions drawn by previous researchers on the need of considering and treating vulnerabilities differently from defects (Canfora et al. 2020; Camilo et al. 2015; Joshi et al. 2015; Mercaldo et al. 2018; Morrison et al. 2018; Russo et al. 2019). On the other hand, our results indicate that the same refactoring can have multiple, contrasting effects on code quality and dependability.

### Implications of the Study

The results of our study provide us with several actionable items and implications for both researchers and practitioners that we discuss in the following.

**Novel Refactoring Optimization Techniques.**: According to our results, refactoring is generally not connected to software vulnerabilities. However, we pointed out that refactoring operations dealing with generalization can contribute to the improvement of software systems’ security profile under certain perspectives. By connecting the previous research on the effect of refactoring on defects introduction with the results of our study, we could conclude that the definition of novel strategies that recommend refactoring operations—while minimizing the negative impact on source code attributes—should be devised and further investigated. In particular, the key example is represented by search-based refactoring recommendations (Mariani and Vergilio 2017; O’Keeffe and Cinnéide 2008), where search-based algorithms are used to recommend developers the best refactoring operation (or sequence of operations) to apply based on the potential impact that such a refactoring may have on various properties of source code. These recommenders might be potentially enhanced by means of the addition of further security-related objective functions so that they could recommend refactoring operations that optimize the compromise between quality and security metrics/technical debt. For instance, let us consider the case of *Extract Superclass*, which we found to appear among the most disruptive refactorings for security according to our results. The refactoring consists of finding a subset of methods of a class that can be extracted in order to create a new superclass. As such, there are multiple ways to perform the refactoring based on how the subset of methods to extract is identified. An *Extract Superclass* refactoring recommender might use, as an objective function, a weighted combination of quality and security metrics so that it can identify the subset of methods to extract that optimize both quality and security. Similarly, multi-objective search algorithms might be used to solve the problem, for instance by combining information coming from quality metrics, security metrics, and technical debt. Some preliminary studies on these aspects have been recently published (Abid et al. 2020), yet we believe that further studies are needed, especially concerning the granularity of the recommendations. Indeed, our **RQ**_3_ shows that developers would benefit from just-in-time solutions that can provide advice while committing new changes to software repositories.
**Exploiting Security Variations to Drive Refactoring.**: The results coming from **RQ**_1_ and **RQ**_2_ also pointed out the existence of refactoring operations having high correlations with both increase or decrease of security metrics and technical debt. In these cases, our findings suggest that the positive or negative effect on security is due to the specific operation performed when refactoring code. In a real-case scenario, these results may be exploited to devise automated mechanisms that alert developers of the potential effects of refactoring on security. As an example, we may envision the definition of novel bots/conversational agents (Alizadeh et al. 2019) that monitor the development and drive the developer toward the application of an operation that has higher chances to improve security metrics or reduce security technical debt when recognizing he/she is applying a refactoring operation. The research in this respect is rapidly gaining interest (Erlenhov et al. 2019; Lebeuf et al. 2017; Beschastnikh et al. 2017), though actionable solutions are still not widely spread, and so representing a potentially interesting use case.
**Refactoring Verification and Validation.**: As a complimentary discussion of the previous one, we can foresee two main implications for the testing community. First and foremost, the importance of having robust verification and validation techniques is further corroborated by our study. Practitioners and security managers can indeed exploit our findings to put in place additional preventive mechanisms aimed at verifying the outcome of each modification, possibly improving both the code review process (Pascarella et al. 2018), e.g., by integrating stricter security checks when refactoring operations are applied, and the regression testing activities (Wong et al. 1997) of their systems. Secondly, our results shed light on the need for more research on techniques to verify the correctness of refactoring operations. This is an overly neglected angle of the refactoring process (Soares et al. 2010) that has been only tangentially touched by the research community in the past (Bladel and Demeyer 2018; Soares et al. 2012). We hope that our investigation would stimulate research on this topic.
**Homogenizing Refactoring Operations.**: As noticed in **RQ**_1_, some refactoring operations, e.g., *Extract Superclass*, tend to have different effects for security depending on how they are applied. This finding—which we believe would deserve further attention—possibly suggests that practitioners approach refactoring in different manners, perhaps because of their different expertise or level of knowledge on the classes subject to refactoring. As such, they could benefit from automated solutions that can recommend *how* to apply the refactoring, namely what are the steps that may lead to the safe improvement of source code quality and homogenize the refactoring process toward the definition of standard guidelines that might favor both newcomers and developers with limited knowledge on security.
**The Link between Composite and Elementary Refactorings**.: In this empirical study we investigated the effect of two composite refactoring operations, namely *Extract & Move Method* and *Move & Inline Method*. We suspected that they might behave differently from the isolated application of the basic refactoring operations they are composed of—i.e., *Extract Method*, *Move Method*, and *Inline Method*. The results of **RQ**_1_ show that composite refactoring operations appear to behave as if they are a “mixture” of their basic operations; conversely, in **RQ**_2_ we observed that they have some effects on a restricted subset of SonarQube violations, while their basic operations do not. Similarly, in **RQ**_3_ *Extract & Move Method* tends to occur more often in vulnerability-contributing commits than individual *Extract Method* and *Move Method* refactorings. Based on these results, we could not outline a precise trend regarding composite refactorings. In any case, the number of instances in our dataset was limited, hence demanding further in-depth investigations with a larger number of observations to derive more precise conclusions on how these composite refactoring operations are connected to their individual refactoring operations.
**Value of The Currently Available Security Metrics.**: When collecting the data required to address **RQ**_1_, we observed that the security metrics previously proposed in the literature (Abid et al. 2020; Alshammari et al. 2010b; Agrawal and Khan 2014) capture similar aspects, being all computed based on the number of security-sensitive attributes that a class exposes. We consider it a limitation that does not enable a comprehensive analysis of the source code’s security profile. As such, a side outcome of our study suggests that more effort should be invested in the definition of novel security metrics that may adequately complement the analysis of the attributes. This represents a challenge for the software engineering community and researchers in closely related fields, e.g., programming languages, which are called to elicit specific properties that make programming languages more or less prone to security weaknesses.
**Refactoring Has A Poor Impact on Security Technical Debt**.: From the results achieved in the context of **RQ**_2_, we could observe that most refactoring operations do not significantly vary the amount of security-related technical debt. The *Extract Superclass* and *Extract Interface* refactoring types represent exceptions to this discussion, along with composite refactoring operations. As such, we can claim that refactoring is mostly safe with respect to security technical debt, yet verification and validation mechanisms might represent useful additions to assess the refactored code against security regressions.
**Software Vulnerabilities: A Social Perspective?**: The results given by our statistical modeling exercise revealed that, in most cases, the projects themselves turn out to significantly influence the increase/decrease of security-related metrics and technical debt. While this aspect deserves ad-hoc investigations to better understand the underlying reasons leading to these findings, our study seems to suggest that there exist specific properties or standards implemented within those projects that have effects on software dependability. In other words, our results seem to be in line with recent studies uncovering relations between developer’s collaboration/coordination—elaborated and controlled through the definition of development contribution guidelines (Elazhary et al. 2019) and code of conducts (Tourani et al. 2017)—and the implications they have for software quality (Palomba et al. 2018; Kwan et al. 2011). In this sense, our results can serve as a base for investigations into the role of social aspects on vulnerabilities.

## Threats to Validity

Several factors might have biased our results. This section discusses them and reports the mitigation strategies we employed.

### Construct Validity

The subjects of our study were the commits having refactoring operations, that we could detect using the tool RefactoringMiner (Tsantalis et al. 2018). The main threat associated with this granularity level is the impossibility to isolate the refactored code elements and study how their security profile has changed. Despite the fact that RefactoringMiner allows the identification of the refactored code regions, we still had trouble in selecting a reasonable set of metrics capturing the security profiles at such a granularity level. In other words, there are no metrics that can measure the security of partial code snippets: the minimum unit of work is the file/class. Nevertheless, we strove for addressing at our best this issue by removing the amount of noise from refactoring commits—whenever the metrics and tools allowed. Specifically, the computation of the security metrics (**RQ**_1_), the number of violations (**RQ**_2_), and the confounding variables (both **RQ**_1_ and **RQ**_2_) did not involve the files not subject to any of the refactoring operations occurred in a commit. Unfortunately, we could not do the same for the security remediation effort metric (**RQ**_2_) as the tool SonarQube is only able to compute it at the entire project’s snapshot level. In spite of everything, this mitigation mechanism still does not exclude any form of changes unrelated to refactorings. Currently, this is the best possible solution to the best of our knowledge.

In the context of **RQ**_1_ and **RQ**_2_, we employed automated tools to compute security metrics and technical debt. As for the security metrics, we re-implemented the tool by Abid et al. (2020) as it was not publicly available. When developing Surface, we followed the exact steps reported in Abid et al. (2020), other than conducting follow-up automated and manual testing sessions to assess the results produced by the tool. For the sake of verifiability, we made Surface publicly available in our online appendix (Iannone et al. 2022). Among the technical debt detectors available in the literature, the selection of SonarQube was driven by the results reported by Saarimaki et al. (2019), who showed that it is accurate when considering security violations. Moreover, these tools were supported by the libraries PyDriller (Spadini et al. 2018) and Lizard to facilitate the recovery of the change history and the computation of the confounding variables (LOC, WCM, and code churn), respectively. Both are widely applied in several software repository mining studies.

We expressed the security-related metrics for the commits by aggregating the deltas we computed on all the files directly involved in refactorings. In particular, the metrics CA (Classified Attributes) and CM (Classified Methods) were summed, while the rest of the metrics were averaged. CA and CM count the number of security-sensitive code components (attributes or methods), so the sum suits well to count the amount of changed security-sensitive code components within the commit. On the contrary, all the other security metrics ranged between 0 and 1, expressing “no exposure” to “maximal exposure”, respectively. Despite the existence of other aggregators, such as the median, we opted to use the average as it well summarizes the change in the exposure levels of all the refactored files without reducing the effect of outliers (i.e., sharp changes in the security metrics).

As for **RQ**_3_, our results might have been affected by the erroneous identification of vulnerability-fixing and vulnerability-contributing commits. In the first case, we mined the fixing commits from the references reported in the CVE records description in the National Vulnerability Database (NVD). Despite being considered a reliable source of information that is continuously monitored and updated, we cannot exclude the case in which the CVE record fails at reporting the entire set of patches—indeed, an insufficient set of fixing commits would have reduced the amount of contributing commits our algorithm fetched.

In the second case, we employed a set of heuristics built on top of SZZ (Sliwerski et al. 2005) to recover the VCCs. While the performance of the algorithm has been criticized in the past (Rodríguez-Pérez et al. 2018a), a recent study (Rosa et al. 2021) has shown that (i) the performance of SZZ depends on the dataset to which it is applied and (ii) the original version of SZZ is the one providing the best performance, overall. Moreover, it has been seen as one of the best possible strategies for recovering VCCs. For this reason, we were careful to adopt all possible recommended precautions to greatly reduce the amount of noise and improve the precision, e.g., ignoring irrelevant files, blaming the context of blocks of new code, etc. To be even more confident about the suitability of our VCCs mining algorithm, we manually validated its results, observing a precision of 71%, which we considered acceptable for our purposes. Lastly, our strategy is also robust to most cases of files renamings. As a matter of fact, the git-blame functionality can automatically detect file renamings when traversing the project’s history, further reducing the risk of blaming wrong commits.

### Internal Validity

When building statistical models in **RQ**_1_ and **RQ**_2_, we selected three confounding factors, i.e., LOC, WMC, and code churn, to control our findings for aspects that might have explained the (in)stability of security metrics and violations better than the number of instances of refactoring operations. We acknowledge the existence of additional factors that were not considered in our study, and, as such, replications of our work would be desirable. Nonetheless, our manual follow-up analysis (see Section 4.1) had the goal of further investigating the underlying reasons behind the relation between refactoring and vulnerabilities, possibly mitigating threats to internal validity and also explaining the role of confounding factors on our results.

Different implementations of refactoring operations might affect the level of security of source code differently or may even represent explicit compromises between quality and security made by a developer. For example, a *Pull Up Attribute* refactoring typically leads to a visibility change of a private attribute: this might be either performed by modifying the visibility into protected or public so that the attribute can be accessible by child classes. While the protected visibility would be essential to apply the refactoring, the public visibility might potentially induce unnecessary risks for security—unless developers consciously opt for this choice and favor it because of other contextual factors or requirements. In this respect, it is worth remarking that our empirical study does not aim at questioning the way developers may apply refactoring, but rather what effect refactoring types may have on the security profile of source code. Furthermore, the specific design decisions taken by a developer when performing refactoring cannot be automatically detected through the refactoring mining tools currently available. Therefore, we encourage replications of our study conducted with different research methods, e.g., through controlled experiments that verify how the refactoring choices done by developers impact security.

### Conclusion Validity

Concerning the relation between treatment and outcome, a threat is related to the statistical methods adopted to address our **RQ** s. In **RQ**_1_ and **RQ**_2_, we opted for a Multinomial Log-Linear statistical model (Theil 1969) as our problem was a multiclass problem involving both categorical and continuous independent variables. In addition, it allowed us to interpret the results from various perspectives, i.e., by considering both statistical codes and odds ratios. Before interpreting the results, we also verified the normality of the independent variable distributions through the Anderson-Darling normality test (Anderson and Darling 1952) before computing the Spearman’s rank-correlation coefficients (Spearman 1961) to identify pairs of correlated independent variables that might lead to multicollinearity.

### External Validity

Our study targeted 39 projects from the *Technical Debt Dataset* (Lenarduzzi et al. 2019) involving 7,708 commits containing refactorings. While almost all the systems belong to the Apache Software Foundation, they were originally selected to meet guidelines that ensure diversity and representativeness (Nagappan et al. 2013; Patton 2002). We cannot exclude that different results could be obtained when considering systems of other ecosystems developed using different programming languages and with different maturity levels. In addition, it is worth remarking that **RQ**_3_ could only target nine of those projects, namely the ones connected to the NVD dataset of known vulnerabilities. Replications targeting a larger set of projects would be, therefore, desirable. In any case, in our online appendix (Iannone et al. 2022), we made available the data and scripts to favor researchers interested in replicating our study in other contexts.

## Related Work

The impact of refactoring on source code dependability has been explored from different perspectives, which we overview herein.

### Impact of Refactoring on Software Quality

Many studies have investigated the impact of refactoring on software quality either directly or from the perspective of defect proneness, change proneness, or code smells.

Bavota et al. (2015) mined the history of 63 releases of Java Open Source Projects (OSPs) to investigate refactoring operations on code components, which indicate a need for refactoring through indicators such as metrics and smells. They concluded that most refactoring operations take place on code with no quality metrics indication for the need to refactor, and although 40% of refactoring operations were performed on smelly code, only 7% removed the smells. Their findings were corroborated by Yoshida et al. (2016) who revisited the relationship between code smells and refactoring by using the same refactoring dataset by Bavota et al. (2015). Cedrim et al. (2017) had similar findings when they analyzed more than 16K+ refactoring instances from 23 OSPs to investigate whether refactoring reduces the code smell density. They reported that even though almost 80% of refactorings touched smelly code, 57% of refactorings did not impact them, and roughly 10% of them removed the smells while 33% introduced new smells. Tufano et al. (2017) conducted an empirical study on 200 projects from the Android, Apache, and Eclipse ecosystems to investigate, among other aspects, whether developers’ actions, e.g., refactoring, resolve smells. They reported that only a low number (9%) of code smells are removed following refactoring operations.

Palomba et al. (2017) investigated the relationship between refactoring operations and code changes (namely fault repairing modification, general maintenance modification, and feature introduction modification) by analyzing the dataset by Bavota et al. (2015). They concluded that code duplication and Self-Admitted Technical Debt (SATD) are the main reasons behind refactoring instances, and refactoring also helps increase code readability. The impact of refactoring on code readability was the subject of the study by Sellitto et al. (2022), who partially confirmed previous findings, showing that refactoring can also negatively impact code readability metrics.

Kim et al. (2012) investigated the refactoring benefits and challenges at Microsoft by conducting a survey, semi-structured interviews, and historical data analysis. They found that refactoring is beneficial, leading to reduced inter-module dependencies and post-release defects. An empirical study was conducted by Bavota et al. (2012) to investigate the impact of refactoring on defects. They found that generally, refactoring instances do not induce defects. However, in specific cases, some specific refactoring types (e.g., Pull Up Method and Extract Subclass) tend to introduce defects in code.

### Impact of Refactoring on Software Security

Mumtaz et al. (2018) investigated whether removing code smells through refactoring resulted in improved security for a system. They conducted a study to identify a subset of code smells and calculated security metrics on five systems. Then they applied refactoring to remove the smells and then re-calculated the same metrics as before. They concluded that generally, refactoring improved the quality of the studied systems from a security standpoint. Ghaith and Cinnéide (2012) were interested in finding out whether automated search-based refactoring improved software security. They achieved an improvement of 15% in the metrics of industrial software after applying search-based refactoring. However, their study is based on a small project, and the results are not generalizable.

An empirical study was conducted by Abid et al. (2020) to determine the relationship between quality and security and the impact of refactoring types on security. The results of the study were used to implement a tool, which was then evaluated on OSPs. They concluded that their tool improved the security of the systems with little impact on the quality. They further validated their results by conducting a survey with practitioners. Similarly, Alshammari et al. (2010a) assessed the impact of refactoring at the design level on security using a case study. Their findings indicate that about 20 refactoring rules improve security, 12 rules made security worse, and four rules had no impact on security. In a follow-up study (Alshammari et al. 2012), the authors evaluated the impact of refactoring on information security using a case study. 8 out of 16 refactoring rules used improved the software’s security while the remaining made it worse. Again, both studies being focused on one case study cannot be generalized. Maruyama and Omori (2011) proposed a tool, implemented as an Eclipse plug-in, to help developers assess the impact of their refactoring operations on software vulnerabilities during software implementation. Currently, the tool supports only two refactoring types, namely, *Pull Up Method* and *Push Down Method*, and measure security using access levels (private, public, protected, and default) of fields. A downgrade in the access level signifies that the software becomes more vulnerable. However, they evaluated the tool using an artificial experiment (on one version of Eclipse) and not on real software.

### Impact of Refactoring on Security-Related Technical Debt

Refactoring has been recognized in many studies as one of the most common ways to manage technical debt (Pérez et al. 2020; Codabux and Williams 2013; Codabux et al. 2014). In this subsection, we review some studies to understand the impact of refactoring on security debt (TD).

Zabardast et al. (2020) investigated the impact of various software development activities, including refactoring on TD by analyzing 2K+ commits in a large industrial project. Their empirical study shows that refactoring removes 22% of TD but introduces an additional 22% TD. However, in most cases, refactoring did not impact TD. Search-based automated refactoring using four different approaches was used by Mohan et al. (2016) to determine the impact of refactoring on TD, among other aspects of development, on six OSPs. They concluded that automated refactoring help decrease TD in software. However, these studies do not focus on security-related TD. Similarly, there are some studies on security smells, which are symptoms in the code that signals the prospect of a security vulnerability (Ghafari et al. 2017). Such studies investigate whether the security smells have an impact on vulnerabilities and are conducted in specific domains (Ghafari et al. 2017; Rahman et al. 2019).

### Reflecting on Previous Work and Our Contribution

To summarize, the studies that investigate the impact of refactoring on software quality have mixed results. Some reported a positive impact whereas others concluded that refactoring increased TD or had no impact on it. Most existing studies focus on the impact of refactoring on software quality but very few investigate TD specifically. Similarly, the studies which investigate the impact of refactoring on software security do not include security-related technical debt. The studies are also limited, often focusing on one software system, thereby making their results not generalizable. Despite refactoring being commonly used to reduce technical debt, most existing research focuses on code smell as an indication of debt, thereby explaining the lack of refactoring studies that focus on technical debt directly. Similarly, security smells have been investigated in the context of vulnerabilities but not refactoring. Abid et al. (2020) conducted a preliminary study to investigate the impact of refactoring types on security but, to the best of our knowledge, investigating the actual impact of refactoring on technical debt from a security standpoint has not been studied and therefore represents a premier of our research.

## Conclusion

The potential adverse effects of refactoring on software dependability have been previously assessed concerning its relation to software defects (Bavota et al. 2012; Di Penta et al. 2020). In this study, we went a step forward by considering the extent to which refactoring affects software security. We have conducted a three-level analysis that considered the effects of refactoring on (i) security metrics, (ii) security-related technical debt, and (iii) contribution to the introduction of known vulnerabilities. Our study had a primarily quantitative connotation where we employed statistical methods on a set of 39 open-source projects. Yet, we conducted additional manual analyses to extract qualitative insights and possible motivations explaining the statistical findings. The core results of the study reported that refactoring has a limited impact on security. Nevertheless, some exceptions indicate that some particular types of refactoring operations might lead to significant variations of software systems’ security profiles under different perspectives. Particularly interesting, in this respect, was the case of refactoring operations dealing with the generalization that appeared to disrupt the source code security.

Based on our findings, we identified several open issues and challenges for researchers, especially related to the lack of automated mechanisms to balance multiple dependability attributes. These outcomes represent our future research agenda, which is focused on the definition of novel just-in-time vulnerability detectors, technical debt linters, and testing methods to verify the presence and exploitability of software vulnerabilities. Additionally, we plan to extend the study by considering a more comprehensive range of software projects, refactoring operations (e.g., “big” or architectural refactoring (Martin and Kent 1999)), and security-related indicators (e.g., security smells (Ghafari et al. 2017)), other than triangulating our findings with different research methods, e.g., through controlled studies able to reveal how different refactoring implementations may lead to a variation of software security indicators.

## Data Availability

The datasets built during the current study, plus the scripts used to analyze and generate the data, are available in the FigShare repository: https://figshare.com/articles/online_resource/Rubbing_Salt_in_the_Wound_A_Large-Scale_Investigation_into_the_Effects_of_Refactoring_on_Vulnerabilities/14483787/1.

## Footnotes

1 https://github.com/iNPUTmice/Conversations

2 https://github.com/apache/batik

3 Link: https://www.sonarqube.org

4 Given our study and currently available standards, we followed the general guidelines when reporting the study design and results.

5 Link: https://github.com/tsantalis/RefactoringMiner

6 Link: https://rules.sonarsource.com

7 https://pypi.org/project/lizard

8 https://cran.r-project.org/web/packages/nnet/nnet.pdf

9 https://pypi.org/project/lizard

## References

[CR1] Abid C, Kessentini M, Alizadeh V, Dhouadi M, Kazman R (2020) How does refactoring impact security when improving quality? a security-aware refactoring approach. IEEE Transactions on Software Engineering

[CR2] Adèr H. J (2008) Advising on research methods: A consultant’s companion. Johannes van Kessel Publishing

[CR3] Agrawal A, Khan R (2014) Assessing impact of cohesion on security-an object oriented design perspective. Pensee 76(2)

[CR4] Al Dallal J, Abdin A (2017). Empirical evaluation of the impact of object-oriented code refactoring on quality attributes: a systematic literature review. IEEE Trans Softw Eng.

[CR5] Alhazmi O, Malaiya Y, Ray I (2007). Measuring, analyzing and predicting security vulnerabilities in software systems. Comput Secur.

[CR6] Alizadeh V, Kessentini M, Mkaouer MW, Ocinneide M, Ouni A, Cai Y (2018). An interactive and dynamic search-based approach to software refactoring recommendations. IEEE Trans Softw Eng.

[CR7] Alizadeh V, Ouali MA, Kessentini M, Chater M (2019) RefBot: Intelligent software refactoring bot. In: 2019 34th IEEE/ACM international conference on automated software engineering (ASE). IEEE, pp 823–834

[CR8] Alshammari B, Fidge C, Corney D (2009) Security metrics for object-oriented class designs. In: International conference on quality software. IEEE, pp 11–20

[CR9] Alshammari B, Fidge C, Corney D (2010) Assessing the impact of refactoring on security-critical object-oriented designs. In: Asia pacific software engineering conference. IEEE, pp 186–195

[CR10] Alshammari B, Fidge C, Corney D (2010) Security metrics for object-oriented designs. In: Australian software engineering conference. IEEE, pp 55–64

[CR11] Alshammari B, Fidge C, Corney D (2012) Security assessment of code refactoring rules. In: National workshop on information assurance research, VDE, pp 1–10

[CR12] Anderson TW, Darling DA (1952). Asymptotic theory of certain “Goodness of Fit” criteria based on stochastic processes. Ann Math Stat.

[CR13] Avgeriou P, Taibi D, Ampatzoglou A, Arcelli Fontana F, Besker T, Chatzigeorgiou A, Lenarduzzi V, Martini A, Moschou N, Pigazzini I, Saarimäki N, Sas D, Soares de Toledo S, Tsintzira A (2021) An overview and comparison of technical debt measurement tools. IEEE Software

[CR14] Azeem MI, Palomba F, Shi L, Wang Q (2019). Machine learning techniques for code smell detection: a systematic literature review and meta-analysis. Inf Softw Technol.

[CR15] Bavota G, De Carluccio B, De Lucia A, Di Penta M, Oliveto R, Strollo O (2012) When does a refactoring induce bugs? an empirical study. In: 12th international working conference on source code analysis and manipulation. IEEE, pp 104–113

[CR16] Bavota G, De Lucia A, Di Penta M, Oliveto R, Palomba F (2015). An experimental investigation on the innate relationship between quality and refactoring. J Syst Softw.

[CR17] Bavota G, De Lucia A, Marcus A, Oliveto R (2014). Automating extract class refactoring: an improved method and its evaluation. Empir Softw Eng.

[CR18] Beschastnikh I, Lungu MF, Zhuang Y (2017) Accelerating software engineering research adoption with analysis bots. In: 2017 IEEE/ACM 39th international conference on software engineering: New ideas and emerging technologies results track (ICSE-NIER). IEEE, pp 35–38

[CR19] Bladel BV, Demeyer S (2018) Test behaviour detection as a test refactoring safety. In: International workshop on refactoring, pp 22–25

[CR20] Bland JM, Altman DG (2000). The odds ratio. BMJ.

[CR21] The MITRE Corporation (2023a) Common vulnerabilities and exposures. Accessed 16 February 2023, https://cve.mitre.org/

[CR22] The MITRE Corporation (2023b) Cve search tool. Accessed 16 February 2023, https://github.com/cve-search/cve-search

[CR23] Camilo F, Meneely A, Nagappan M (2015) Do bugs foreshadow vulnerabilities? a study of the chromium project. In: 12th working conference on mining software repositories. IEEE, pp 269–279

[CR24] Canfora G, Di Sorbo A, Forootani S, Pirozzi A, Visaggio CA (2020). Investigating the vulnerability fixing process in OSS projects: Peculiarities and challenges. Comp Sec.

[CR25] Cedrim D, Garcia A, Mongiovi M, Gheyi R, Sousa L, de Mello R, Fonseca B, Ribeiro M, Chávez A (2017) Understanding the impact of refactoring on smells: A longitudinal study of 23 software projects. In: Joint meeting on foundations of software engineering, pp 465–475

[CR26] Chidamber SR, Kemerer CF (1994). A metrics suite for object oriented design. IEEE Trans Softw Eng.

[CR27] Chowdhury I, Zulkernine M (2011). Using complexity, coupling, and cohesion metrics as early indicators of vulnerabilities. J Syst Archit.

[CR28] Codabux Z, Williams B (2013) Managing technical debt: An industrial case study. In: International workshop on managing technical debt (MTD). IEEE, pp 8–15

[CR29] Codabux Z, Williams BJ, Niu N (2014) A quality assurance approach to technical debt. In: International conference on software engineering research and practice (SERP)

[CR30] Cohen J (2013). Statistical power analysis for the behavioral sciences.

[CR31] Curtis B, Sappidi J, Szynkarski A (2012) Estimating the size, cost, and types of technical debt. In: 2012 3rd international workshop on managing technical debt (MTD). IEEE, pp 49–53

[CR32] Di Penta M, Bavota G, Zampetti F (2020) On the relationship between refactoring actions and bugs: a differentiated replication. In: Joint meeting on european software engineering conference and symposium on the foundations of software engineering, pp 556–567

[CR33] de Paulo Sobrinho EV, DeLucia A, de Almeida Maia M (2018) A systematic literature review on bad smells—5 w’s: which, when, what, who, where. IEEE Transactions on Software Engineering

[CR34] El Emam K, Benlarbi S, Goel N, Rai SN (2001). The confounding effect of class size on the validity of object-oriented metrics. IEEE Trans Softw Eng.

[CR35] Elazhary O, Storey M-A, Ernst N, Zaidman A (2019) Do as i do, not as i say: Do contribution guidelines match the github contribution process?. In: International conference on software maintenance and evolution (ICSME), pp 286–290

[CR36] Erlenhov L, de Oliveira Neto FG, Scandariato R, Leitner P (2019) Current and future bots in software development. In: 2019 IEEE/ACM 1st international workshop on bots in software engineering (BotSE). IEEE, pp 7–11

[CR37] Ghafari M, Gadient P, Nierstrasz O (2017) Security smells in android. In: The 17th international working conference on source code analysis and manipulation (SCAM), pp 121–130

[CR38] Ghaith S, Cinnéide MÓ (2012) Improving software security using search-based refactoring. In: International symposium on search based software engineering. Springer, pp 121–135

[CR39] Goyal PK, Joshi G (2014) QMOOD metric sets to assess quality of java program. In: International conference on issues and challenges in intelligent computing techniques (ICICT). IEEE, pp 520–533

[CR40] Huang S, Tang H, Zhang M, Tian J (2010) Text clustering on national vulnerability database. In: International conference on computer engineering and applications, vol 2, pp 295–299

[CR41] Iannone E, Codabux Z, Lenarduzzi V, De Lucia A, Palomba F (2022) Rubbing salt in the wound? a large-scale investigation into the effects of refactoring on security. [Online]. Accessed 16 February 2023. Available: https://figshare.com/articles/online_resource/Rubbing_Salt_in_the_Wound_A_Large-Scale_Investigation_into_the_Effects_of_Refactoring_on_Vulnerabilities/14483787/1 10.1007/s10664-023-10287-x PMC10209315 37250850

[CR42] Iannone E, Guadagni R, Ferrucci F, De Lucia A, Palomba F (2022) The secret life of software vulnerabilities: a large-scale empirical study. IEEE Trans Softw Eng :1–1

[CR43] Joshi C, Singh UK, Tarey K (2015). A review on taxonomies of attacks and vulnerability in computer and network system. Int J.

[CR44] Kim M, Zimmermann T, Nagappan N (2012) A field study of refactoring challenges and benefits. In: 20th international symposium on the foundations of software engineering, pp 1–11

[CR45] Kim M, Zimmermann T, Nagappan N (2014). An empirical study of refactoringchallenges and benefits at microsoft. IEEE Trans Softw Eng.

[CR46] Koru AG, Liu H (2005) An investigation of the effect of module size on defect prediction using static measures. In: Workshop on Predictor models in software engineering, pp 1–5

[CR47] Kutner MH, Nachtsheim CJ, Neter J, Li W (2005). Applied linear statistical models, vol 5.

[CR48] Kwan I, Schroter A, Damian D (2011). Does socio-technical congruence have an effect on software build success? a study of coordination in a software project. IEEE Trans Softw Eng.

[CR49] Lebeuf C, Storey M-A, Zagalsky A (2017). Software bots. IEEE Softw.

[CR50] Lenarduzzi V, Saarimäki N, Taibi D (2019) The technical debt dataset. In: 15th conference on PREdictive Models and data analycs In Software Engineering, ser. PROMISE ’19, pp 2–11

[CR51] Mann HB, Whitney DR (1947). On a test of whether one of two random variables is stochastically larger than the other. Ann Math Stat.

[CR52] Mariani T, Vergilio SR (2017). A systematic review on search-based refactoring. Inf Softw Technol.

[CR53] Martin F, Kent B (1999). Refactoring: Improving the design of existing code.

[CR54] Maruyama K, Omori T (2011) A security-aware refactoring tool for java programs. In: Workshop on Refactoring Tools, pp 22–28

[CR55] McCabe TJ (1976). A complexity measure. IEEE Trans Softw Eng.

[CR56] McGraw G (2004). Software security. IEEE Secur Priv.

[CR57] Meneely A, Srinivasan H, Musa A, Tejeda A, M Mokary, Spates B (2013) When a patch goes bad: Exploring the properties of vulnerability-contributing commits. In: International symposium on empirical software engineering and measurement, pp 65–74

[CR58] Mens T, Tourwé T (2004). A survey of software refactoring. IEEE Trans Softw Eng.

[CR59] Mercaldo F, Di Sorbo A, Visaggio CA, Cimitile A, Martinelli F (2018). An exploratory study on the evolution of android malware quality. J Softw Evol Proc.

[CR60] Mohan M, Greer D, McMullan P (2016). Technical debt reduction using search based automated refactoring. J Syst Softw.

[CR61] Morrison PJ, Pandita R, Xiao X, Chillarege R, Williams L (2018). Are vulnerabilities discovered and resolved like other defects?. Empir Softw Eng.

[CR62] Mumtaz H, Alshayeb M, Mahmood S, Niazi M (2018). An empirical study to improve software security through the application of code refactoring. Inf Softw Technol.

[CR63] Murphy-Hill E, Black AP (2008) Breaking the barriers to successful refactoring: observations and tools for extract method. In: International conference on Software engineering, pp 421–430

[CR64] National Institute for Standards (2023) U.S. NIST computer security division. Accessed 16 February 2023, https://www.nist.gov

[CR65] National Institute for Standards (2023) National vulnerability database. Accessed 16 February 2023, https://nvd.nist.gov/

[CR66] Nagappan N, Ball T (2005) Use of relative code churn measures to predict system defect density. In: International conference on Software engineering, pp 284–292

[CR67] Nagappan M, Zimmermann T, Bird C (2013) Diversity in software engineering research. In: 2013 9th joint meeting on foundations of software engineering, pp 466–476

[CR68] O’Keeffe M, Cinnéide MO (2008). Search-based refactoring for software maintenance. J Syst Softw.

[CR69] Palomba F, Tamburri DA, Arcelli Fontana F, Oliveto R, Zaidman A, Serebrenik A (2018) Beyond technical aspects: How do community smells influence the intensity of code smells? IEEE Trans Softw Eng :1–1

[CR70] Palomba F, Zaidman A, Oliveto R, De Lucia A (2017) An exploratory study on the relationship between changes and refactoring. In: 25th international conference on program comprehension (ICPC). IEEE, pp 176–185

[CR71] Pascarella L, Spadini D, Palomba F, Bruntink M, Bacchelli A (2018) Information needs in contemporary code review. In: Proceedings of the ACM on human-computer interaction, vol. 12, no. CSCW, pp. 1–27

[CR72] Patton M (2002). Qualitative evaluation and research methods.

[CR73] Per R, Martin H (2009). Guidelines for conducting and reporting case study research in software engineering. Empirical Softw Engg.

[CR74] Pérez B, Castellanos C, Correal D, Rios N, Freire S, Spínola R, Seaman C (2020) What are the practices used by software practitioners on technical debt payment: results from an international family of surveys. In: International conference on technical debt, pp 103–112

[CR75] Perl H, Dechand S, Smith M, Arp D, Yamaguchi F, Rieck K, Fahl S, Acar Y (2015) Vccfinder: Finding potential vulnerabilities in open-source projects to assist code audits. In: Proceedings of the 22nd ACM SIGSAC conference on computer and communications security, ser. CCS ’15. Association for Computing Machinery, New York, pp 426–437. 10.1145/2810103.2813604

[CR76] Rahman A, Parnin C, Williams L (2019) The seven sins: Security smells in infrastructure as code scripts. In: International conference on software engineering (ICSE). IEEE, pp 164–175

[CR77] Ralph P, bin Ali N, Baltes S, Bianculli D, Diaz J, Dittrich Y, Ernst N, Felderer M, Feldt R, Filieri A, de França BBN, Furia CA, Gay G, Gold N, Graziotin D, He P, Hoda R, Juristo N, Kitchenham B, Lenarduzzi V, Martínez J, Melegati J, Mendez D, Menzies T, Molleri J, Pfahl D, Robbes R, Russo D, Saarimäki N, Sarro F, Taibi D, Siegmund J, Spinellis D, Staron M, Stol K, Storey M-A, Taibi D, Tamburri D, Torchiano M, Treude C, Turhan B, Wang X, Vegas S (2021) Empirical standards for software engineering research

[CR78] Rodríguez-Pérez G, Robles G, González-Barahona J (2018). Reproducibility and credibility in empirical software engineering: a case study based on a systematic literature review of the use of the SZZ algorithm. Inf Softw Technol.

[CR79] Rodríguez-Pérez G, Robles G, González-Barahona JM (2018). Reproducibility and credibility in empirical software engineering: A case study based on a systematic literature review of the use of the SZZ algorithm. Inf Softw Technol.

[CR80] Rosa G, Pascarella L, Scalabrino S, Tufano R, Bavota G, Lanza M, Oliveto R (2021) Evaluating SZZ implementations through a developer-informed oracle. In: International conference on software engineering (ICSE). p. to appear. IEEE

[CR81] Russo ER, Di Sorbo A, Visaggio CA, Canfora G (2019). Summarizing vulnerabilities’ descriptions to support experts during vulnerability assessment activities. J Syst Softw.

[CR82] Saarimaki N, Baldassarre MT, Lenarduzzi V, Romano S (2019) On the accuracy of SonarQube technical debt remediation time. In: Euromicro conference on software engineering and advanced applications (SEAA). IEEE, pp 317–324

[CR83] Sellitto G, Iannone E, Codabux Z, Lenarduzzi V, De Lucia A, Palomba F, Ferrucci F (2022) Toward understanding the impact of refactoring on program comprehension. In: 2022 IEEE international conference on software analysis, evolution and reengineering (SANER), pp 731–742

[CR84] Sharma T, Suryanarayana G, Samarthyam G (2015). Challenges to and solutions for refactoring adoption: an industrial perspective. IEEE Softw.

[CR85] Shin Y, Meneely A, Williams L, Osborne JA (2010). Evaluating complexity, code churn, and developer activity metrics as indicators of software vulnerabilities. IEEE Trans Softw Eng.

[CR86] Shin Y, Williams L (2008) An empirical model to predict security vulnerabilities using code complexity metrics. In: International symposium on Empirical software engineering and measurement, pp 315–317

[CR87] Silva D, Silva J, Santos GJDS, Terra R, Valente MTO (2020) Refdiff 2.0: A multi-language refactoring detection tool. IEEE Transactions on Software Engineering

[CR88] Silva D, Tsantalis N, Valente MT (2016) Why we refactor? Confessions of github contributors. In: 24th ACM sigsoft international symposium on foundations of software engineering, pp 858–870

[CR89] Sliwerski J, Zimmermann T, Zeller A (2005) When do changes induce fixes?. In: International workshop on mining software repositories, ser. MSR ’05, pp 1–5

[CR90] Soares G, Gheyi R, Massoni T (2012). Automated behavioral testing of refactoring engines. IEEE Trans Softw Eng.

[CR91] Soares G, Gheyi R, Serey D, Massoni T (2010). Making program refactoring safer. IEEE Softw.

[CR92] Spadini D, Aniche M, Bacchelli A (2018) PyDriller: Python framework for mining software repositories. In: Proceedings of the 2018 26th ACM joint meeting on european software engineering conference and symposium on the foundations of software engineering - ESEC/FSE 2018. ACM Press, New York, pp 908–911. [Online]. Available: http://dl.acm.org/citation.cfm?doid=3236024.3264598

[CR93] Spearman C (1961) The proof and measurement of association between two things 10.1093/ije/dyq191 21051364

[CR94] Stroggylos K, Spinellis D (2007) Refactoring–does it improve software quality?. In: International workshop on software quality (WoSQ’07: ICSE Workshops 2007). IEEE, pp 10–10

[CR95] Sukamolson S (2007). Fundamentals of quantitative research. Lang Inst Chulalongkorn Univ.

[CR96] Terra R, Valente MT, Miranda S, Sales V (2018). JMove: a novel heuristic and tool to detect move method refactoring opportunities. J Syst Softw.

[CR97] Theil H (1969). A multinomial extension of the linear logit model. Int Econ Rev.

[CR98] Tourani P, Adams B, Serebrenik A (2017) Code of conduct in open source projects. In: 2017 IEEE 24th international conference on software analysis, evolution and reengineering (SANER). IEEE, pp 24–33

[CR99] Tsantalis N, Chatzigeorgiou A (2009). Identification of move method refactoring opportunities. IEEE Trans Softw Eng.

[CR100] Tsantalis N, Mansouri M, Eshkevari LM, Mazinanian D, Dig D (2018) Accurate and efficient refactoring detection in commit history. In: 40th international conferenc on software engineering, ser ICSE ’18, pp 483–494

[CR101] Tufano M, Palomba F, Bavota G, Oliveto R, Di Penta M, De Lucia A, Poshyvanyk D (2017). When and why your code starts to smell bad (and whether the smells go away). IEEE Trans Softw Eng.

[CR102] Vassallo C, Palomba F, Gall HC (2018) Continuous refactoring in CI: A preliminary study on the perceived advantages and barriers. In: International conference on software maintenance and evolution (ICSME). IEEE, pp 564–568

[CR103] Vassallo C, Panichella S, Palomba F, Proksc S, Gall HC, Zaidman A (2019) How developers engage with static analysis tools in different contexts. Empirical Software Engineering

[CR104] Wong WE, Horgan JR, London S, Agrawal H (1997) A study of effective regression testing in practice. In: International symposium on software reliability engineering. IEEE, pp 264–274

[CR105] Yang L, Li X, Yu Y (2017) Vuldigger: A just-in-time and cost-aware tool for digging vulnerability-contributing changes. In: GLOBECOM 2017 - 2017 IEEE global communications conference, pp 1–7

[CR106] Yoshida N, Saika T, Choi E, Ouni A, Inoue K (2016) Revisiting the relationship between code smells and refactoring. In: International conference on program comprehension (ICPC). IEEE, pp 1–4

[CR107] Zabardast E, Gonzalez-Huerta J, Šmite D (2020) Refactoring, bug fixing, and new development effect on technical debt: An industrial case study. In: Euromicro conference on software engineering and advanced applications (SEAA). IEEE, pp 376–384

[CR108] Zhang H (2009) An investigation of the relationships between lines of code and defects. In: International conference on software maintenance. IEEE, pp 274–283

[CR109] Zhang S, Caragea D, Ou X (2011) An empirical study on using the national vulnerability database to predict software vulnerabilities. In: International conference on database and expert systems applications, pp 217–231

